# PpARF6 acts as an integrator of auxin and ethylene signaling to promote fruit ripening in peach

**DOI:** 10.1093/hr/uhad158

**Published:** 2023-07-31

**Authors:** Xiaomei Chen, Yudi Liu, Xian Zhang, Beibei Zheng, Yuepeng Han, Ruo-Xi Zhang

**Affiliations:** CAS Key Laboratory of Plant Germplasm Enhancement and Specialty Agriculture, Wuhan Botanical Garden, The Innovative Academy of Seed Design of Chinese Academy of Sciences, Wuhan 430074, China; University of Chinese Academy of Sciences, 19A Yuquanlu, Beijing 100049, China; CAS Key Laboratory of Plant Germplasm Enhancement and Specialty Agriculture, Wuhan Botanical Garden, The Innovative Academy of Seed Design of Chinese Academy of Sciences, Wuhan 430074, China; University of Chinese Academy of Sciences, 19A Yuquanlu, Beijing 100049, China; CAS Key Laboratory of Plant Germplasm Enhancement and Specialty Agriculture, Wuhan Botanical Garden, The Innovative Academy of Seed Design of Chinese Academy of Sciences, Wuhan 430074, China; University of Chinese Academy of Sciences, 19A Yuquanlu, Beijing 100049, China; CAS Key Laboratory of Plant Germplasm Enhancement and Specialty Agriculture, Wuhan Botanical Garden, The Innovative Academy of Seed Design of Chinese Academy of Sciences, Wuhan 430074, China; Hubei Hongshan Laboratory, Wuhan 430070, China; CAS Key Laboratory of Plant Germplasm Enhancement and Specialty Agriculture, Wuhan Botanical Garden, The Innovative Academy of Seed Design of Chinese Academy of Sciences, Wuhan 430074, China; Hubei Hongshan Laboratory, Wuhan 430070, China; CAS Key Laboratory of Plant Germplasm Enhancement and Specialty Agriculture, Wuhan Botanical Garden, The Innovative Academy of Seed Design of Chinese Academy of Sciences, Wuhan 430074, China; Hubei Hongshan Laboratory, Wuhan 430070, China

## Abstract

Although auxin is known to induce ethylene biosynthesis in some Rosaceae fruit crops, the mechanisms underlying the auxin–ethylene interaction during fruit ripening remain largely unknown. Here, the regulatory role of an auxin response factor, PpARF6, in fruit ripening was investigated in peach. Peach fruits showed accelerated ripening after treatment with auxin and PpARF6 was found to be significantly induced. PpARF6 not only could induce ethylene synthesis by directly activating the transcription of ethylene biosynthetic genes, but also competed with EIN3-binding F-box proteins PpEBF1/2 for binding to ethylene-insensitive3-like proteins PpEIL2/3, thereby keeping PpEIL2/3 active. Moreover, PpARF6 showed an interaction with PpEIL2/3 to enhance the PpEIL2/3-activated transcription of ethylene biosynthetic genes*.* Additionally, ectopic overexpression of *PpARF6* in tomato accelerated fruit ripening by promoting the expression of genes involved in ethylene synthesis and fruit texture. In summary, our results revealed a positive regulatory role of PpARF6 in peach fruit ripening via integrating auxin and ethylene signaling.

## Introduction

Peach (*Prunus persica*) is an important economic crop that is widely cultivated in temperate regions of the world. Peach fruit exhibits a typical climacteric rise in respiration during ripening, which is accompanied by a dramatic increase in ethylene production and rapid softening of the flesh [1]. Peach cultivars have a great variation in fruit texture, the combination of multiple traits, such as firmness, crunchiness, and meltiness, that directly influence consumer behavior and postharvest storage life. According to fruit texture, peach cultivars are classified as melting (MF), non-melting (NMF), or stony hard (SH). The MF/NMF flesh trait is controlled by an endopolygalacturonase gene cluster within the *freestone-melting flesh* (*F-M*) locus on chromosome (Chr) 4 [2], while the recessive SH phenotype is associated with the causal *PpYUC11* gene in the *hd* locus on Chr 6 [3, 4]. *PpYUC11* encodes a YUCCA (YUC) flavin monooxygenase responsible for the conversion of indole-3-pyruvic acid (IPA) to indole-3-acetic acid (IAA), the main active auxin [5], and its expression in the SH fruit is disrupted by a 2569-bp transposon insertion in the 5′-flanking region [6]. Both MF and NMF cultivars show increased ethylene production and decreased flesh firmness during fruit ripening, although their degrees differ [7]. In contrast, the SH cultivar produces little or no ethylene throughout fruit development and its fruits remain firm and crunchy even when fully ripe [8, 9].

Ethylene biosynthesis involves two enzymes, ACC synthase (ACS) and ACC oxidase (ACO). ACS converts *S*-adenosyl-l-methionine (SAM) to 1-aminocyclopropane-1-carboxylate acid (ACC), while ACO catalyzes the oxidation of ACC to ethylene. MADS-box and NAC transcription factors (TFs) are two of the best-characterized regulators of ethylene synthesis in tomato fruit [10–12]. Moreover, the downstream regulators of the ethylene signaling pathway characterized in *Arabidopsis thaliana*, such as ethylene insensitive3-like (EIL) and ethylene response factor (ERF), are known to play crucial roles in ethylene production in several other species. For example, MdERF2 and MdERF3 in apple regulate ethylene synthesis by suppressing and promoting the expression of *MdACS1*, respectively [13], whereas, MdEIL1 controls ethylene production through the MdEIL1–MdMYB1–MdERF3 module [14]. In cultivated banana, MaERF9 and MaERF11 function as a transcriptional activator or repressor of ethylene biosynthesis genes [15], while MaEIL5 may be involved in ethylene synthesis through interaction with MaNAC1/2 during fruit ripening [16, 17]. In peach, PpERF4 can induce transcription of *PpACO1* to promote fruit ripening [18], while PpEIL1–3 are able to activate transcription of both *PpACS1* and *PpACO1* [19]. Notably, EIL TFs are regulated at the post-translational level in response to the ethylene signal. In the absence of ethylene, EILs are degraded via the ubiquitin proteasome pathway driven by EIN3-binding F-box proteins 1/2 (EBF1/2). However, the EBF1/2-mediated EIL proteolysis is repressed in the presence of ethylene, which causes an accumulation of EILs in the nucleus to induce transcription of *ERF* and other ethylene-responsive genes [20].

While the role of ethylene in fruit ripening has been extensively investigated, the contribution of other plant hormones remains largely elusive. In peach, identification of the causal gene for the SH phenotype demonstrates that IAA synthesis via the IPA–YUC pathway is essential for ethylene production in peach fruit [3, 6]. Moreover, auxin has been reported to induce ethylene biosynthesis in climacteric and non-climacteric fruits [21–23]. Therefore, auxin is assumed to have an intense interplay with ethylene during fruit ripening. The auxin signaling pathway contains three major components, auxin-binding TIR1/AFB F-box proteins, auxin response factor (ARF) TFs, and auxin-induced repressor (Aux/IAA) proteins. When auxin levels are low, Aux/IAA proteins form heterodimers with ARFs to prevent them from binding to TGTCTC auxin-response elements (AuxREs) in promoters of auxin response genes. When auxin levels are high, auxin stimulates Aux/IAA degradation through the ubiquitin/proteasome pathway. Loss of Aux/IAA repressor allows ARF TFs to activate transcription of auxin response genes [24–26].

The importance of ARF and Aux/IAA TFs in regulating auxin-mediated fruit ripening processes has been reported in a range of fruit species. In tomato, SlARF2 manipulates fruit ripening process via regulating ethylene biosynthesis genes such as *SlACO1* and *SlACS2/4* [27], while SlERF.B3 interacts with *SlIAA27* to mediate ethylene–auxin crosstalk [28]. In apple, MdARF5 is capable of initiating fruit ripening through induction of ethylene biosynthetic genes, including *MdERF2*, *MdACS1*, and *MdACO1* [29]. In papaya, a mediator in the auxin–ethylene interaction, CpARF2, interacts with CpEIL1 to promote the CpEIL1-mediated transcription of ethylene biosynthetic genes, leading to an acceleration of fruit ripening [30]. In peach, the role of ARF TFs in fruit ripening remains largely unclear [31]. Overall, the mechanisms by which auxin signaling interplays with ethylene to regulate the fruit ripening process at the transcriptional and post-transcriptional levels are largely unknown.

The objective of this study was to investigate the role of ARF-mediated auxin signaling in fruit ripening of peach. We found that peach fruits treated with 1-naphthaleneacetic acid (NAA) showed accelerated ripening. An NAA-inducible TF termed PpARF6 was identified and it interacted with PpEIL2/3 to integrate auxin and ethylene signals, thereby promoting fruit ripening. Our results provide insight into the molecular mechanisms by which the cross-talk between ethylene and auxin participates in the regulation of fruit ripening in peach.

## Results

### Identification of *PpARF6* putatively involved in regulating peach fruit ripening

**Figure 1:**
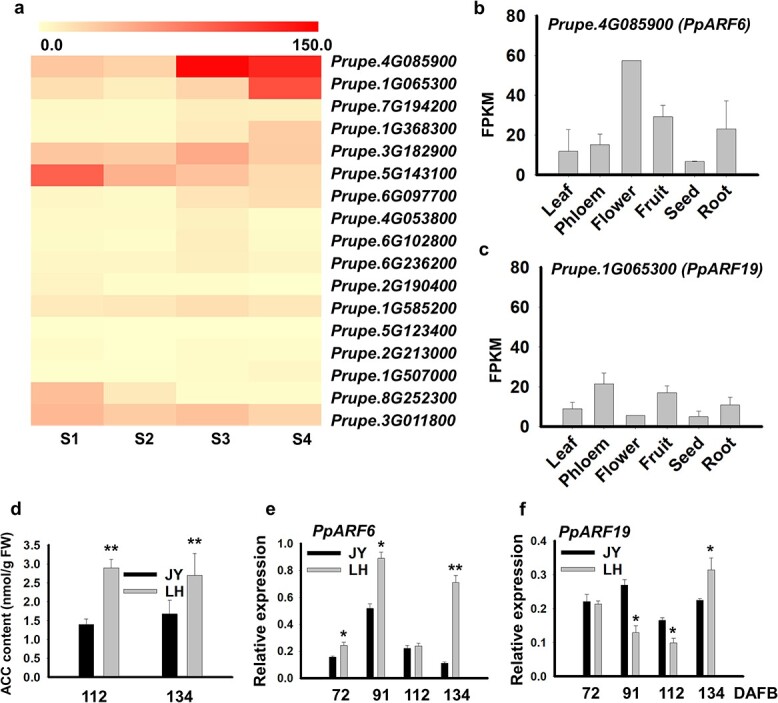
Identification of fruit ripening-related *ARF* TFs in peach. **a** Heat-map analysis of 17 ARF TFs in fruits of MF cultivar ‘XH’ throughout fruit development*.* Expressions of *PpARF6* (Prupe.4G085900) and *PpARF19* (Prupe.1G065300) are dramatically increased during fruit ripening. The S1–S4 stages represent 34, 50, 75, and 117 DAFB, respectively. **b** Expression profile of *PpARF6* in the phloem tissue and various organs, including flower, fruit, root, and seed. (c) Expression profile of *PpARF19* in phloem tissue and various organs, including flower, fruit, root, and seed. **d** ACC content in fruits of two cultivars at different stages of ripeness. **e** RT–qPCR analysis of the *PpARF6* expression in fruits of two cultivars at different stages of ripening. **f** Expression of *PpARF19* in fruits of two cultivars at different stages of ripening. JY, SH cv. ‘Jingyu’; LH, MF cv. ‘Lvhua’ 9. The phases of fruits of JY and LH at 72, 91, 112, and 134 DAFB corresponded to S1, S2, S3, and S4, respectively. ^*^P < .05, ^**^P < .01.

A previous study revealed 17 genes putatively encoding ARF protein in the peach genome [31]. To identify candidate ARF TFs for peach fruit ripening, we analyzed their expression profiles in fruits of MF cultivar ‘XH’ throughout development based on transcriptome sequencing data reported in our previous study [32]. As a result, two *ARF* genes, *PpARF6* (*Prupe.4G085900*) and *PpARF19* (*Prupe.1G065300*), showed a dramatic increase in expression level during ripening stages (Fig. 1a, Supplementary Data Table S3). Strikingly, transcription levels of *PpARF6* were much higher than those of *PpARF19* at late stages of fruit development. We investigated expression profiles of *PpARF6* and *PpARF19* in various samples of the same cultivar based on transcriptome data in a previous study [33]. *PpARF6* was highly expressed in reproductive organs such as ripe fruit and flower (Fig. 1b, Supplementary Data Table S4), while *PpARF19* was predominantly expressed in phloem and ripe fruit (Fig. 1c, Supplementary Data Table S4). Similar to their expression profiles in fruits of ‘XH’ at ripening stages, *PpARF6* showed higher expression in ripe fruit than did *PpARF19* (Fig. 1b and c).

‘Lvhua 9’ (LH), also known as ‘Yanhong’, is an MF peach cultivar, while ‘Jingyu’ (JY) is the SH type [34]. The fruits of both JY and LH reached maturity at 140 days after full bloom (DAFB). The ACC content was significantly lower in fruits of JY at 112 and 134 DAFB compared with those of LH (Fig. 1d). We validated the expression profiles of *PpARF6* and *PpARF19* in fruits of JY and LH throughout development using RT–qPCR, and found that *PpARF6* and *PpARF19* were both highly expressed in fruits of the melting cultivar tested at ripening stages. *PpARF6* displayed much higher expression in ripe fruits of the MF cultivar than that in ripe fruits of the SH cultivar (Fig. 1e), while the expression of *PpARF19* in ripe fruits of the MF cultivar was slightly higher than that in ripe fruits of the SH cultivar (Fig. 1f). In addition, phylogenetic analysis revealed that *PpARF6* and *PpARF19* formed a monophyletic group with apple *MdARF5* involved in fruit ripening [29], but were separated from tomato *SlARF2* [27] and papaya *CpARF2* [30], which have been reported to control fruit ripening (Supplementary Data Fig. S1).

Altogether, the above results suggested a more important role of *PpARF6* in regulating fruit ripening. Thus, *PpARF6* was selected for further functional characterization.

### Roles of auxin in promoting fruit ripening and induction of *PpARF6* transcription in peach

**Figure 2:**
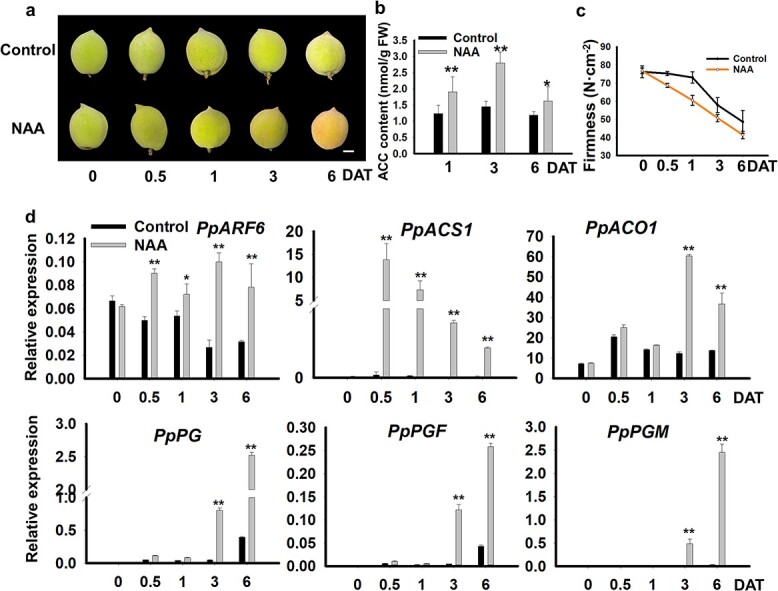
Influence of NAA treatment on fruit ripening of MF peach cultivar ‘Summer Golden’. **a** NAA-treated fruits showed accelerated ripening. Fruits treated with ddH_2_O were used as control. Fruits used for treatment were collected at 60 DAFB. Fruits after treatment were stored in a growth chamber at 25°C under a 12 h/day photoperiod. The white bar indicates 1 cm. **b** ACC content in peach fruits after treatment with NAA. **c** Changes in fruit firmness after NAA treatment. **d** Expression profiles of *PpARF6*, ethylene biosynthetic genes, and softening-related genes in peach fruits after treatment with NAA or ddH_2_O. ^*^P < .05, ^**^P < .01.

The fruits of MF cultivar ‘Summer Golden’ reached maturity at 75 DAFB. To determine whether auxin could induce the transcription of *PpARF6*, we treated immature fruits of ‘Summer Golden’ at 60 DAFB with NAA. Red pigmentation occurred on the surface of NAA-treated fruits at 6 days after treatment (DAT), while no red pigmentation was observed on the surface of ddH_2_O-treated fruits (Fig. 2a). Consistently, ACC content in NAA-treated fruits was significantly higher than in ddH_2_O-treated fruits (Fig. 2b). Firmness values of NAA-treated fruits showed a dramatic decrease at 0.5 and 1 DAT, while a slight decrease was observed for ddH_2_O-treated fruits (Fig. 2c). Additionally, the expression of *PpACS1* in NAA-treated fruits was significantly activated at 0.5 DAT and then showed a decreasing trend (Fig. 2d). However, no significant difference in *PpACO1* expression between NAA- and ddH_2_O-treated fruits was observed until 3 DAT. Later, the expression of *PpACO1* in NAA-treated fruits at 6 DAT showed a dramatic decrease. The transcription levels of softening-related genes in NAA-treated fruits, such as *PpPG*, *PpPGF*, and *PpPGM*, were significantly upregulated at 3 DAT and all showed an increase at 6 DAT. Interestingly, RT–qPCR analysis indicated that the level of *PpARF6* transcripts in NAA-treated fruits was significantly upregulated at 0.5 DAT, with a peak at 3 DAT (Fig. 2d). In addition, fruits of the SH cultivar ‘Xiacui’ at the earlier stage of ripeness were treated with NAA. Ethylene content showed a significant increase after 3 days of treatment (Supplementary Data Fig. S2a), and firmness was dramatically decreased after 1 day of treatment (Supplementary Data Fig. S2b). Consistently, the expression levels of ethylene biosynthetic genes and softening-related genes were significantly increased after NAA treatment (Supplementary Data Fig. S2c and d). These results indicated that the application of exogenous auxin could accelerate peach fruit ripening, and *PpARF6* could be involved in the regulation of auxin-induced fruit ripening.

### Validation of the role of *PpARF6* in promoting peach fruit ripening

**Figure 3:**
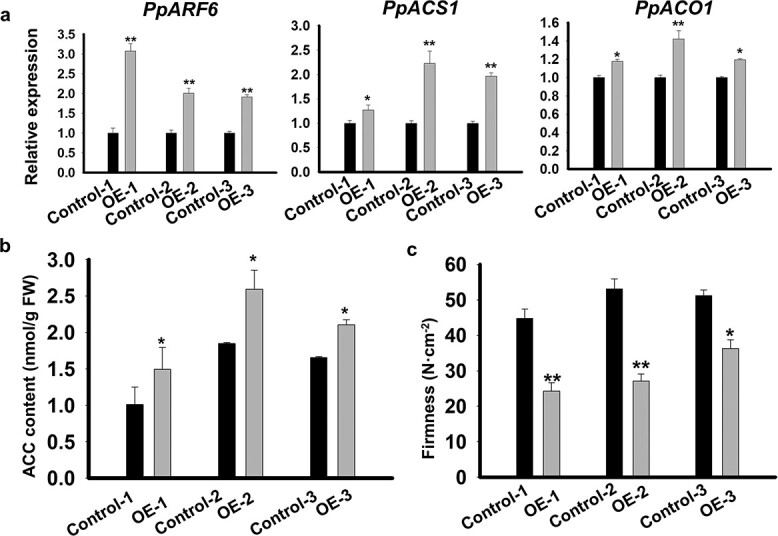
Functional analysis of the regulatory role of *PpARF6* in ethylene synthesis and fruit firmness via transient overexpression assay. **a** Expression of *PpARF6*, *PpACS1*, and *PpACO1* in flesh tissues around the sites infiltrated with *PpARF6*. **b** ACC content in flesh tissues around the sites infiltrated with *PpARF6*. **c** Firmness of fruits infiltrated with *PpARF6*. Expression of *PpARF6* is under the control of the super-promoter. Both gene expression and flesh firmness were determined 3 days after infiltration. ^*^P < .05, ^**^P < .01.

The fruits of melting flesh peach cultivar ‘Dongxuemitao’ reached maturity at 230 DAFB. To verify the role of *PpARF6* in ethylene biosynthesis, we conducted a transient expression assay in fruits of ‘Dongxuemitao’ at 210 DAFB. *Agrobacterium* cultures harboring the *PpARF6* gene driven by the super-promoter were infiltrated into one side of off-tree fruit, while another side of the same fruit was injected with *Agrobacterium* cultures harboring the empty vector as control. Three days after infiltration, the expression level of *PpARF6* was significantly higher in flesh tissues around the sites infiltrated with *PpARF6* than that in those infiltrated with empty vector (Fig. 3a, Supplementary Data Fig. S3a). Of the infiltrated fruits, three with relatively high expression of *PpARF6* were selected to investigate gene expression, ACC content, and firmness (Fig. 3). The expression levels of ethylene biosynthetic genes *PpACS1* and *PpACO1* and ACC content in *PpARF6*-infiltrated fruits were significantly higher than in empty vector infiltrated fruits (Fig. 3a and b). Accordingly, firmness values of *PpARF6*-infiltrated fruits were significantly lower than those of empty vector-infiltrated fruits (Fig. 3c, Supplementary Data Fig. S3b). Moreover, we conducted transient overexpression of *PpARF6* in SH peach ‘Xiacui’ at 100 DAFB (S4 stage). Six days after infiltration, flesh tissues surrounding the sites infiltrated with *PpARF6* displayed increased expression of *PpARF6* (Supplementary Data Fig. S4a), accompanied by a significant increase in ethylene content and a significant decrease in firmness (Supplementary Data Fig. S4b and c). These results indicated the possibility that *PpARF6* is involved in the regulation of ethylene biosynthetic genes in peach fruit.

**Figure 4:**
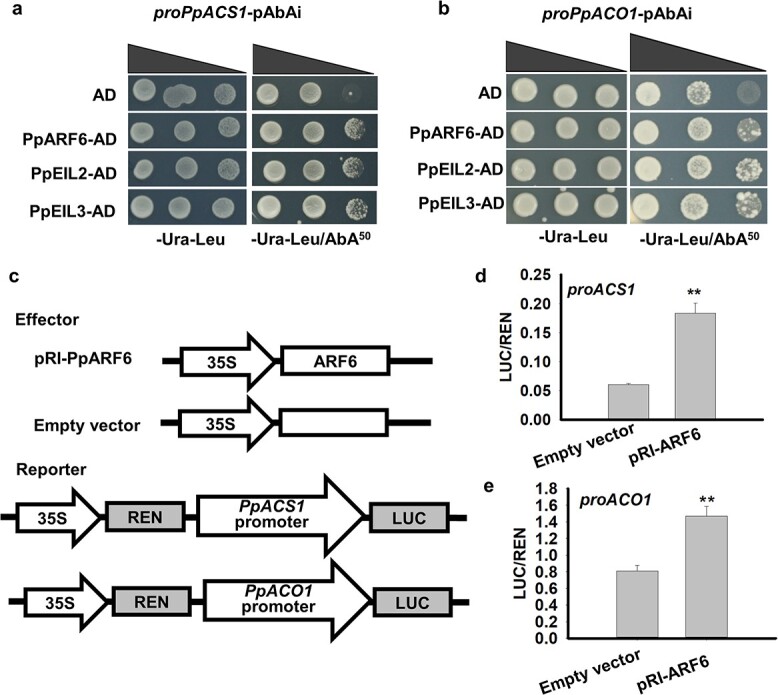
Assay of activating effect of PpARF6 on the promoters of ethylene biosynthetic genes. **a**, **b** Assessment of interaction of PpARF6 and PpEIL2/3 with the *PpACS1* or *PpACO1* promoter using Y1H. The AbA concentration in −Ura−Leu/AbA^50^ medium was 50 ng/ml. **c** Effector and reporter used in the transient dual-luciferase (LUC/REN) assay. **d**, **e** Assessment of activation of PpARF6 on the promoters of *PpACS1* and *PpACO1* using the transient dual-luciferase reporter assay. Transcriptional activation activity was measured 3 days after infiltration, and each treatment contained at least three biological replicates. ^**^P < .01.

ARF TFs are known to bind to AuxREs with the consensus sequences TGTCAN (A/T/C/G) and TGTCNN (TT/TC/GA/GG/CA) [35]. Three and four AuxREs were identified in the promoter sequences of *PpACS1* and *PpACO1* based on PlantCARE analysis (Supplementary Data Fig. S5). To validate whether PpARF6 could interact with the promoters of *PpACO1* and *PpACS1*, we performed the yeast one-hybrid (Y1H) assay. Yeast cells harboring both the *PpACS1* or *PpACO1* promoter and the *PpARF6* gene could grow on SD/−Ura−Leu medium containing 50 ng/ml aureobasidin A (AbA), indicating that PpARF6 is capable of binding to the *PpACS1* and *PpACO1* promoters (Fig. 4a and b). To further confirm the effect of PpARF6 on the transcription of ethylene biosynthetic genes, we performed the dual-luciferase assay in tobacco leaves, with the effector vector containing the *PpARF6* gene and the reporter gene fused with the *PpACS1* or *PpACO1* promoter (Fig. 4c). The LUC/REN ratios of the empty vector plus either *PpACS1* or *PpACO1* promoter were significantly lower than those of the *PpARF6* gene plus either *PpACS1* or *PpACO1* promoter (Fig. 4d and e). This indicated that *PpARF6* functions as a positive regulator in the transcription of ethylene biosynthetic genes.

**Figure 5:**
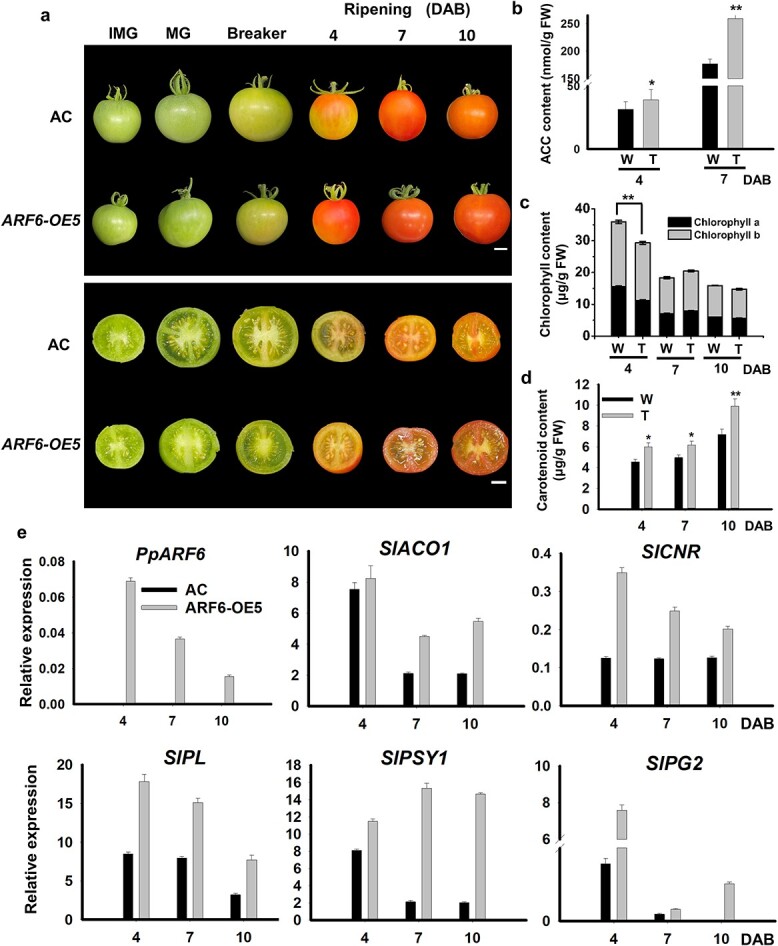
Functional analysis of *PpARF6* through its stable overexpression in tomato. **a** Transgenic fruits overexpressing *PpARF6* throughout development. AC, wild-type control. DAB, days after breaker. **b**–**d** Total ACC, chlorophyll, and carotenoid contents in tomato fruits at different stages of ripening. W, wild-type fruits of AC; T, transgenic fruits overexpressing *PpARF6*. **e** Expression profiles of genes related to ripening, ethylene synthesis and fruit texture in tomato fruits at different stages of ripening. ^*^P < .05, ^**^P < .01.

Finally, the role of *PpARF6* in fruit ripening was confirmed by generating tomato transgenic lines overexpressing *PpARF6* under the super-promoter due to lack of a stable genetic transformation system for peach. Ten stable transgenic lines overexpressing *PpARF6* were obtained and three lines were investigated for fruit ripening behavior (Fig. 5a, Supplementary Data Fig. S6a). The *PpARF6* gene was highly expressed in fruits at ripening stages of these three transgenic lines (Fig. 5e, Supplementary Data Fig. S6b). The color transition from green to red in transgenic fruits was earlier than that in the non-transgenic wild-type control (Fig. 5a, Supplementary Data Fig. S6a), suggesting an acceleration of the ripening process in transgenic fruits. The transgenic line ARF6-OE5 was used as a representative to conduct physiological and molecular characterization of fruits at the following three stages: 4, 7, and 10 days after breaker (DAB). On average, the wild-type fruits reached the breaker stage at 38 days post-anthesis (DPA), while the period from anthesis to breaker stage shortened to 35 DPA in transgenic fruits. The ACC content in transgenic fruits of ARF6-OE5 at 4 and 7 DAB increased by 24.3 and 46.7%, respectively, compared with wild type (Fig. 5b). Since the expression of *SlACO1*, *SlACS2*, and *SlACS4* showed an increase in transgenic tomato fruits (Supplementary Data Figs S6c and S7a and b), we investigated whether PpARF6 could activate tomato ethylene biosynthetic genes. Analysis of *cis* regulatory elements using PlantCARE revealed the presence of AuxREs in the promoters of *SlACO1*, *SlACS2*, and *SlACS4* (Supplementary Data Fig. S7c). The activation of PpARF6 on the promoters of *SlACO1*, *SlACS2* and *SlACS4* was confirmed by the dual-luciferase assay in tobacco leaves (Supplementary Data Fig. S7d).

The amounts of chlorophyll a and b in transgenic fruits of ARF6-OE5 at 4 DAB were significantly lower than in wild-type fruits, but no significant difference was detected at 7 and 10 DAB (Fig. 5c). The accelerated chlorophyll degradation in transgenic fruits suggested a role of *PpARF6* in promoting chlorophyll degradation during late stages of fruit development. Interestingly, the carotenoid content was significantly higher in transgenic fruits of ARF6-OE5 throughout the ripening process than in wild-type fruits (Fig. 5d), suggesting a positive effect of overexpression of *PpARF6* on fruit carotenoid accumulation. The accelerated chlorophyll degradation and increased carotenoid accumulation were also observed in transgenic fruits of ARF6-OE1 and ARF6-OE2 at the ripening stage (Supplementary Data Fig. S6d and e). Additionally, the expression levels of genes associated with fruit ripening and softening were significantly higher in transgenic fruits at 4, 7, and 10 DAB compared with wild-type fruits (Fig. 5e). Notably, transgenic lines overexpressing PpARF6 showed an increase in plant height and stem diameter and exhibited early flowering compared with the wild type (Supplementary Data Fig. S8). Collectively, these results suggested a positive regulatory role of *PpARF6* in vegetative growth and fruit ripening.

### PpARF6, like PpEBF1/2, was able to interact with PpEIL2/3 in peach

**Figure 6:**
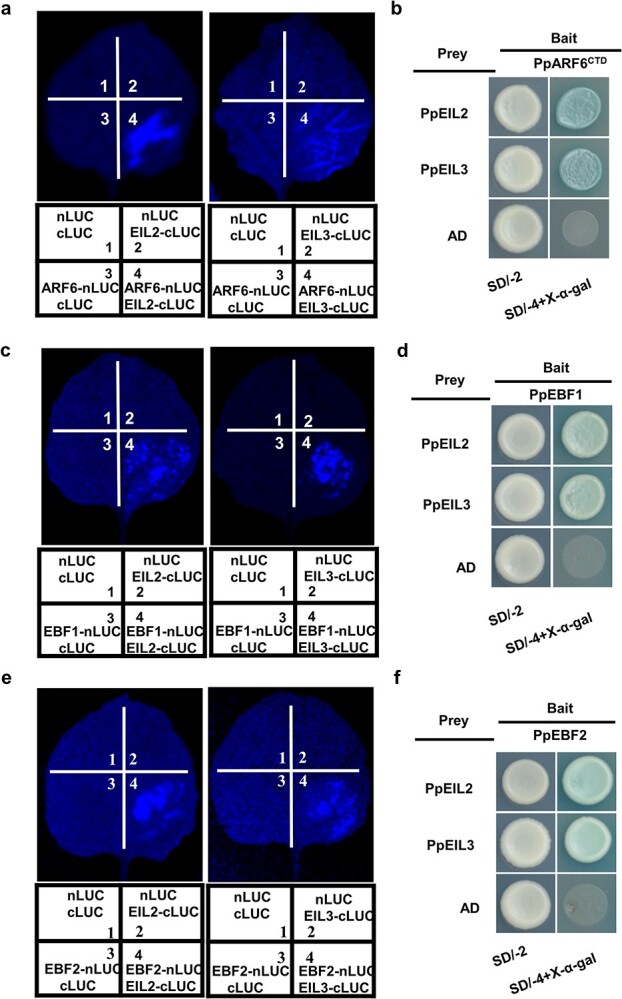
Assay of physical interaction of PpARF6 and PpEBF1/2 with PpEIL2/3 using the firefly NC-LUC assay (**a**, **c**, **e**) in *N. benthamiana* leaves and the Y2H assay (**b**, **d**, **f**). For the firefly NC-LUC assay, PpARF6 and PpEBF1/2 were fused with the N-terminal fragment of firefly luciferase (nLUC), while PpEIL2/3 was fused with the C-terminal fragment of firefly luciferase (cLUC). Empty vector was used as negative control. The numbers represent specific combinations as indicated below. Interaction signals were only detected for the co-transformation of PpEIL2/3 and either PpARF6 or PpEBF1/2. For the Y2H assay, PpARF6 and PpEBF1/2 were fused with BD vector as bait, while PpEIL2 and PpEIL3 were fused with AD vector as prey. Empty vector (AD) was used as control.

As mentioned above, PpARF6 could directly activate the transcription of ethylene biosynthetic genes. To determine whether PpARF6 was able to interact with EIL TF, a master regulator in the ethylene signaling pathway, we performed the firefly luciferase complementation imaging (NC-LUC) assay in tobacco leaves. Our previous study revealed five EIL TFs in the peach genome, with three, termed PpEIL1–3, showing high levels of expression in ripe fruit [19]. Hence, these three PpEILs were tested for their interaction with PpARF6. A luminescence signal was detected for leaf cells expressing both *PpARF6* and either *PpEIL2* or *PpEIL3* (Fig. 6a), while no signal was detected for the interaction of PpEIL1 with PpARF6 (Supplementary Data Fig. S9a)*.* Similarly, the yeast two-hybrid (Y2H) assay indicated that PpEIL1 could not interact with PpARF6 (Supplementary Data Fig. S9b).

EBF proteins are known to interact with EILs, leading to EIL degradation via the ubiquitin proteasome pathway [20]. We identified two *EBF* genes in the peach genome, which showed high similarity to *AtEBF1* and *AtEBF2* and were thus designated *PpEBF1* and *PpEBF2*, respectively. Since *PpEBF1* and *PpEBF2* had high levels of expression in ripe fruit (Supplementary Data Fig. S10, Supplementary Data Table S4), we tested their interaction with PpEILs using the NC-LUC assay. As a result, PpEIL2 and PpEIL3 both could interact with PpEBF1 (Fig. 6c) and PpEBF2 (Fig. 6e). To confirm the results of the NC-LUC assays, we further conducted the Y2H assay. Yeast cells expressing *PpARF6* and either *PpEIL2* or *PpEIL3* could grow and were blue-colored on SD/−Trp−Leu−His−Ade (SD/−4) medium containing X-α-Gal (Fig. 6b), suggesting an interaction between PpARF6 and PpEIL2/3. Similarly, the Y2H revealed that both PpEBF1 and PpEBF2 were able to interact with PpEIL2 and PpEIL3 (Fig. 6d and f). In addition, the assay of subcellular localization revealed that PpARF6, PpEIL2, and PpEIL3 were all located in the nucleus (Supplementary Data Fig. S11), supporting the idea that they function as TFs.

### PpARF6 was capable of weakening the interaction between PpEBF1/2 and PpEIL2/3

**Figure 7:**
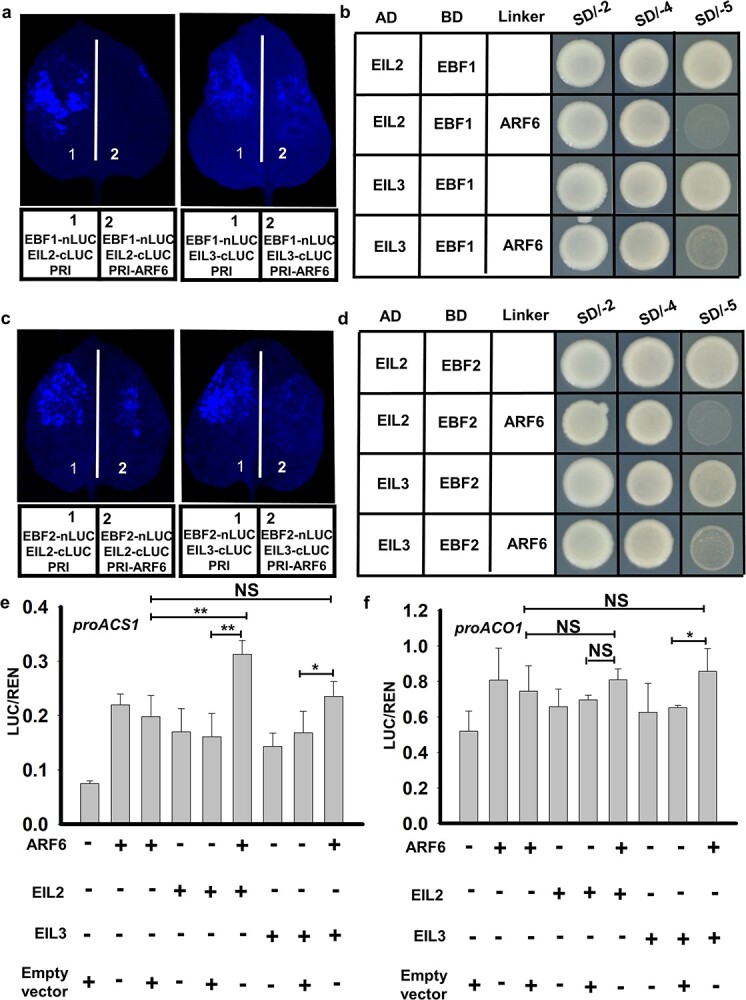
Assessment of competitive binding of PpARF6 to PpEIL2/3 with PpEBF1/2 using the NC-LUC assay (**a**, **c**) and the Y3H assay (**b**, **d**), as well as the effect of PpARF6 on the PpEIL2/3-activated transcription of ethylene biosynthetic genes using the LUC/REN assay (**e**, **f**). For the NC-LUC assay, the luminescence intensity was used to estimate the competitive binding of PpARF6 to PpEIL2/3 with PpEBF1. For the Y3H assay, yeast cells carrying PpEBF1/2 and PpEIL2/3 could grow on SD/−5 medium, while yeast cells carrying PpARF6, PpEBF1/2, and PpEIL2/3 could not. For the LUC/REN assay, PpARF6 and PpEIL2/3 effectors were co-infiltrated into tobacco leaves with the *PpACS1* or *PpACO1* reporter. *P < .05, **P < .01.

To investigate the possibility that PpARF6 competes with PpEBF1/2 for binding to PpEIL2/3, we performed the NC-LUC assay. A strong luminescence signal was observed for tobacco leaves infiltrated with both PpEBF1 and either PpEIL2 or PpEIL3, whereas the luminescence intensity was greatly reduced when PpARF6 was co-infiltrated with both PpEBF1 and either PpEIL2 or PpEIL3 (Fig. 7a). Similarly, co-infiltration of PpEBF2 with either PpEIL2 or PpEIL3 resulted in a strong luminescence signal, but the intensity of the luminescence signal was decreased when PpARF6 was co-infiltrated with both PpEBF2 and either PpEIL2 or PpEIL3 (Fig. 7c). These results suggested that PpARF6 could impair the interaction of PpEBF1/2 with PpEIL2/3. To confirm this finding, we carried out the yeast three-hybrid (Y3H) assay. The results indicated that yeast cells carrying PpEBF1 and either PpEIL2 or PpEIL3 could grow on the SD/−4 medium, while yeast cells carrying PpARF6, PpEBF1, and either PpEIL2 or PpEIL3 could not grow on SD/−Trp−Leu−His−Ade−Met (SD/−5) medium (Fig. 7b). Likewise, yeast cells carrying PpEBF2 and either PpEIL2 or PpEIL3 could grow on the SD/−4 medium, while yeast cells carrying PpARF6, PpEBF2, and either PpEIL2 or PpEIL3 could not grow on the SD/−5 medium (Fig. 7d). The Y3H results confirmed that PpARF6 has an antagonistic effect on the interaction between PpEBF1/2 and PpEIL2/3.

EIL binding sites were identified in the promoter sequences of *PpACS1* and *PpACO1* based on the PlantCARE analysis (Supplementary Data Fig. S5). Y1H assay showed that both PpEIL2 and PpEIL3 could bind to the promoters of *PpACS1* (Fig. 4a) and *PpACO1* (Fig. 4b). Moreover, the dual-luciferase assay indicated that both PpEIL2 and PpEIL3 were able to activate transcription of *PpACS1* (Fig. 7e) and *PpACO1* (Fig. 7f). Then, the synergistic effect of PpARF6 and PpEIL2/3 on the transcription of ethylene biosynthesis genes was validated using the dual-luciferase assay in tobacco leaves. The activation activity of PpEIL2 and PpEIL3 on the *PpACS1* promoter was significantly increased when PpARF6 was co-infiltrated with either PpEIL2 or PpEIL3 (Fig. 7e). Similarly, the activation activity of PpEIL3 on the *PpACO1* promoter was significantly increased when PpARF6 was co-infiltrated with PpEIL3 (Fig. 7f). However, co-infiltration of PpEIL2 with PpARF6 showed no synergistic effect on *PpACO1* transcription compared with the infiltration of PpEIL2 alone. In addition, co-infiltration of PpARF6 and PpEIL2 significantly increased *PpACS1* transcription compared with the infiltration of PpARF6 alone, while a slight but no significant increase was detected for co-infiltration of PpARF6 with PpEIL3 (Fig. 7e). Co-infiltration of PpARF6 with either PpEIL2 or PpEIL3 had no significant increased effect on *PpACO1* transcription compared with the infiltration of PpARF6 alone (Fig. 7f). Collectively, these results suggested that PpARF6 could keep PpEIL2/3 active through competing with PpEBF1/2 for binding to them, thereby enhancing the PpEIL2/3-activated transcription of ethylene biosynthetic genes.

## Discussion

In climacteric fruits, the ripening process is accompanied by a burst of ethylene production that occurs in an autocatalytic manner, in contrast to a basal auto-inhibitory level during development prior to ripening. Auxin has been thought to act as an ethylene antagonist that delays the fruit ripening process [36, 37]. However, a growing body of research demonstrates that auxin accumulation is concurrent with the burst of ethylene during the ripening of climacteric fruits, such as peach [21], plum [38], and apple [39]. The disruption of auxin biosynthesis inhibits the induction of autocatalytic ethylene production in SH peach fruit during ripening [3, 6]. Nevertheless, exogenous auxin application is able to induce autocatalytic ethylene production in SH peach fruit, leading to fruit softening [40]. In this study, we further revealed that an auxin signaling component, PpARF6, functions as a positive regulator of peach fruit ripening through activating genes associated with ethylene biosynthesis. These results were consistent with previous reports [21, 40] and raised the possibility that auxin participates in ripening control via modulating the generation of the climacteric ethylene positive feedback loop.

The transition from the auto-inhibitory feedback loop during fruit development to the autocatalytic feedback loop during the ripening of mature fruit depends on the activation of ethylene biosynthetic genes. In tomato, the upregulation of *ACS2/4* induces climacteric ethylene synthesis at the onset of fruit ripening [37, 41]. Similarly, *PpACS1* shows an increase in expression level during fruit ripening in peach [42], and its inactivation is responsible for the suppression of autocatalytic ethylene production in SH fruit [8, 40]. In this study, *PpACS1* was found to be activated half a day after treatment with NAA, two and half days earlier compared with *PpACO1* (Fig. 2d). Thus, *PpACS1* induction seems to be a rate-limiting step in autocatalytic ethylene production, consistent with the previous finding that ACC synthase is the rate-limiting enzyme in the ethylene biosynthetic pathway [43].

A recent study reveals that the autocatalytic feedback loop is elicited mainly by MADS and NAC TFs during the onset of fleshy fruit ripening [11]. In peach, *PpNAC1* in the *MD* locus with a large effect on mature date is involved in the autocatalytic feedback loop, and its transcription is regulated by PpEIN3/PpEIL TFs [11]. Here, our results further revealed an auxin response gene, *PpARF6*, that could be induced by auxin treatment and could physically interact with PpEIL2/3 (Fig. 6a and b). *PpARF6* was weakly expressed during early stages of fruit development, but its expression increased dramatically in the second exponential growth stage (S3) and remained at high levels until the fruits reached full ripening. Interestingly, a similar expression profile was also observed for peach *PpNAC1* in our previous study [44]. Given that *PpNAC1* acts as the main regulator of peach fruit ripening and its expression profile is similar to that of *PpARF6*, it is worthy of further study to address whether the PpARF6–PpEIL2/3 complex could interact with *PpNAC1* to form a module controlling fruit ripening. Like PpNAC1, both PpARF6 and PpEIL2/3 were found capable of activating transcription of ethylene biosynthetic genes. Notably, PpARF6 could interact with PpEIL2/3 to enhance the PpEIL2/3-activated transcription of ethylene biosynthetic genes (Fig. 7e and f). PpARF6 showed stronger activation of the *PpACS1* promoter than on the *PpACO1* promoter, which is consistent with the above-mentioned finding that *PpACS1* plays a key role in autocatalytic ethylene production. Taken together, these results suggest that PpARF6 integrates ethylene and auxin signaling to regulate fruit ripening in peach. However, it is worth noting that *PpARF19*, besides *PpARF6*, was found to be highly expressed in fruits during ripening in this study. There probably exists functional redundancy in the *ARF* gene family in peach. In addition, ARF TFs function as activators or repressors due to an enrichment of the glutamine residue or the proline, serine, and threonine residues in the variable middle transcriptional regulatory region (MR) [45]. Phylogenetic analysis showed that PpARF6 is closely related to *Arabidopsis* AtARF6 (Supplementary Data Fig. S13a), which functions as an activator [46]. Amino acid alignment indicated that PpARF6, like AtARF6, has an enrichment of glutamine in the MR (Supplementary Data Fig. S13b), which is likely responsible for the activating transcriptional activity of PpARF6.

**Figure 8:**
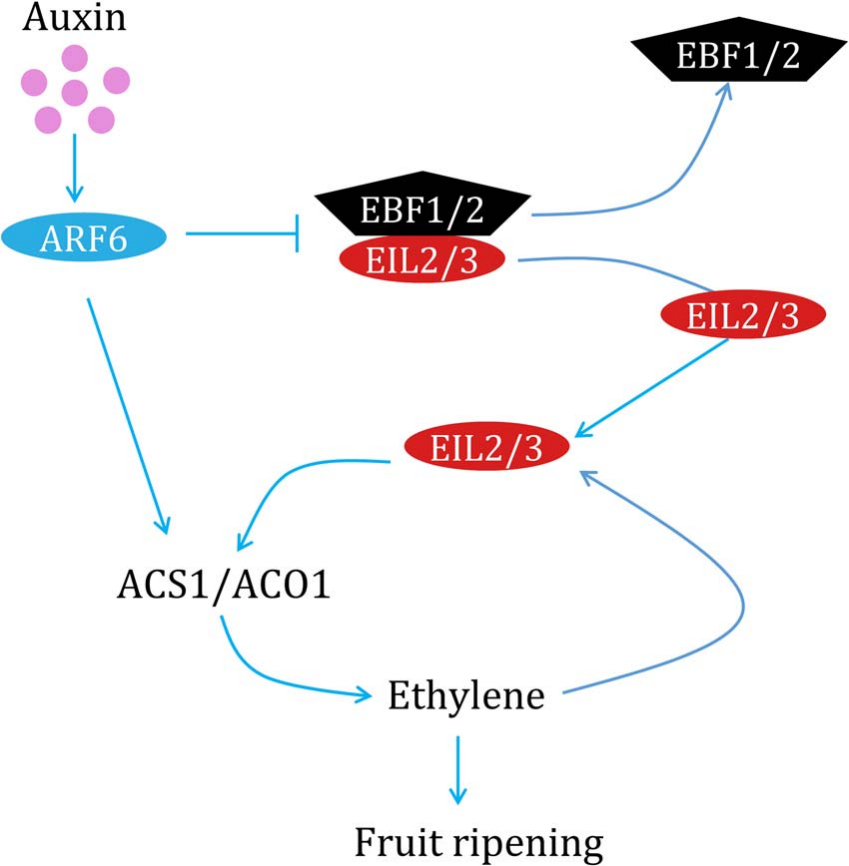
A proposed model for the role of the *PpARF6* gene in promoting fruit ripening. The expression of *PpARF6* in peach fruit is activated upon auxin treatment. PpARF6 can directly activate transcription of ethylene biosynthetic genes. Moreover, PpARF6 competes with PpEBF1/2 for binding to PpEIL2/3, thus keeping PpEIL2/3 active. The activated PpEIL2/3 generates the climacteric ethylene positive feedback loop, leading to fruit ripening.

EILs are known to act as master regulators of the ethylene signaling pathway. However, EIL is an unstable and short-lived nuclear protein [47] as it can be degraded by EBF1/2 proteins through ubiquitin or proteasome-dependent proteolysis [48]. Our previous study indicates that *PpEIL2* and *PpEIL3* are constitutively expressed throughout fruit development [19]. Here, both NC-LUC and Y2H assays demonstrated a strong interaction between PpEBF1/2 and PpEIL2/3 (Fig. 6c–f). Thus, the expression of *PpEIL2/3* in unripe fruit is likely regulated by PpEBF1/2 at the post-translational level to maintain ethylene production at a low basal level during fruit development prior to the onset of ripening. Additionally, the NC-LUC and Y3H assays both indicated that PpARF6 competes with PpEBF1/2 for binding to PpEIL2/3 (Fig. 7a–d), and thus inhibiting dimerization of PpEBF1/2 and PpEIL2/3. The EBF1/2 monomer proteins tend to be sequestered in processing bodies (P-bodies) through binding to the EIN2 C terminal fragment (CEND) [49], or to be degraded through COP1-dependent proteolysis [50]. This will facilitate the accumulation of EILs in the nucleus, thereby promoting the transcription of ethylene biosynthetic genes. Based on the above results, we propose a model for the regulatory role of PpARF6 in fruit ripening via mediating cross-talk between ethylene and auxin in peach (Fig. 8). PpARF6 is activated to directly induce the transcription of ethylene biosynthetic genes upon 200 mg/l auxin treatment, and its activation is also able to inhibit the interaction between PpEBF1/2 and PpEIL2/3, thereby keeping PpEIL2/3 active. The activated PpEIL2/3 will generate the climacteric ethylene positive feedback loop, leading to fruit ripening. Given the finding that disruption of auxin biosynthesis inhibits the induction of autocatalytic ethylene production in peach [3, 6], auxin signaling components such as PpARF6 involved in the cross-talk between auxin and ethylene seem to be crucial for fruit ripening. Like the PpARF6–PpEIL2/3–PpEBF1/2 module, a CpARF2–CpEIL1–CpEBF1 module has been reported to regulate fruit ripening in papaya [30]. However, unlike *PpARF6*, which was induced by auxin, *CpARF2* was inhibited upon auxin treatment. This discrepancy may be partially responsible for the diverse roles of auxin in fruit ripening.

Besides ARF TFs, Aux/IAA proteins play a crucial role in auxin signaling [51]. In this study we investigated the expression of *PpIAA1* in peach fruit treated with NAA. Interestingly, like *PpARF6*, *PpIAA1* was significantly activated half a day after treatment with NAA (Supplementary Data Fig. S12). Thus, it is worthy of further study to ascertain whether and how PpIAA1 plays an important role in integrating auxin and ethylene signals during peach fruit ripening.

It is well known that climacteric ethylene production is associated with fruit softening, chlorophyll degradation and pigment accumulation [52–54]. In peach, the melting flesh trait is mainly controlled by two *PG* genes at the *F-M* locus, *PpPGF* and *PpPGM* [2]. Here, our results showed that the induction of *PpPG* genes at the *F-M* locus occurred 3 days after NAA treatment, while fruit firmness showed a dramatic decrease shortly after treatment with NAA (Fig. 2). These results indicate that in addition to *PpPG* genes at the *F-M* locus with large effect on flesh melting, other genes are involved in regulating fruit texture in peach. Moreover, our results revealed the roles of *PpARF6* in promoting chlorophyll degradation and carotenoid accumulation via its stable overexpression in tomato. Carotenoid accumulation shows an increase in the ripening stages in yellow-fleshed peach fruits [55]. It is worthy of further investigation to address whether *PpARF6* is involved in carotenoid accumulation in peach. Additionally, transgenic tomato lines overexpressing PpARF6 were found to exhibit vigorous vegetative growth (Supplementary Data Fig. S8). Thus, it can be speculated that PpARF6 has a pleiotropic effect on plant growth and development. PpARF6 is phylogenetically related to AtARF6 and AtARF8 (Supplementary Data Fig. S13). In *Arabidopsis*, single mutants of *arf6* and *arf8* display a decrease in plant height and the variation in flower development, while the *arf6 arf8* double mutant exhibits female sterility and a severe dwarf phenotype [56]. Similarly, downregulation of ARF6 and ARF8 in tomato results in shorter petals and stamens as well as decreases in leaf size and internode length [57]. Thus, it seems that the PpARF6 homologs have a conserved function in plant growth and development.

In conclusion, an auxin signaling component, PpARF6, promotes fruit ripening through interacting with ethylene signaling components, PpEILs, to activate transcription of ethylene biosynthetic genes in peach.

## Materials and methods

### Plant materials and fruit treatment

Peach cultivars used in this study, including two SH varieties, ‘Jingyu’ and ‘Xiacui’, and three MF cultivars, ‘Lvhua 9’, ‘Dongxuemitao’, and ‘Summer Golden’, were grown in Wuhan Botanical Garden of the Chinese Academy of Sciences (Wuhan, Hubei province). All peach cultivars were grafted on Maotao rootstock (*P. persica*) and cultivated at a spacing of 4 m × 2 m in 2017. Trees were planted under standard experimental methods of fertilization, pest and disease control, and irrigation. Fruits of ‘Jingyu’ and ‘Lvhua 9’ that were used for RT–qPCR analysis were harvested at 72, 91, 112, and 134 DAFB, corresponding to the following four phases: S1 (the first exponential growth), S2 (pit hardening), S3 (the second exponential growth), and S4 (fruit ripening), respectively. Fruits of ‘Dongxuemitao’ at 210 DAFB (S4) were used for the transient transformation assay. Immature fruits of ‘Summer Golden’ (60 DAFB, S3) and red-colored fruits of ‘Xiacui’ at the earlier stage of ripeness (100 DAFB, S4) were collected for analysis of the effect of treatment with NAA (Phytotechnology Laboratories, Shanghai, China) on fruit ripening. All collected fruits were immediately transferred to the laboratory at room temperature for 3 h and randomly divided into two groups with each containing 50 fruits. One group was soaked in 200 mg/l NAA for 8 min, while another group immersed in ddH_2_O for 8 min was used as control. Following the NAA or ddH_2_O treatment, fruits were stored in the same growth chamber maintained at 25°C with 60–70% relative humidity and a 12-h light/12-h dark photoperiod. The NAA-treated ‘Summer Golden’ fruits were sampled at 0, 0.5, 1, 3, and 6 days post-treatment, while the NAA-treated ‘Xiacui’ fruits were sampled at 0, 1, 3, and 6 days post-treatment. Each sample contained 10 fruits. Flesh firmness was evaluated at two points in the equatorial region of peeled fruit using a GY-4 portable digital fruit hardness tester (Zhejiang Top Instrument, China) equipped with an 8-mm diameter probe. Fruit firmness was recorded in newtons per square centimeter (N cm^−2^). Following firmness measurement, all fruit samples were cut into small pieces, frozen in liquid nitrogen, and stored at −80°C for further experiments.

### RNA extraction and real-time quantitative PCR analysis

Total RNA extraction and the first cDNA strand synthesis were carried out as a previous study [58]. The resulting first-strand cDNA was used as template for RT–qPCR, which was performed as described in our previously reported protocol [59]. *PpTEF2* [60] and *SlACTIN2* [61] were used as the internal control for peach and tomato, respectively. DNA sequences of primers used for RT–qPCR in this study are available in Supplementary Data Table S1.

### Transient overexpression of the *PpARF6* gene in peach fruit

The coding sequence (CDS) of *PpARF6* was cloned into the pSuper1300GFP vector [62]. *Agrobacterium* cells carrying the PpARF6-pSuper1300GFP construct or the empty vector were collected, suspended, and then diluted to an OD_600_ of 0.8 using infiltration buffer (20 mM MES, 10 mM MgCl_2_, 150 mM acetosyringone, pH 5.6). Fruits of MF cultivar ‘Dongxuemitao’ (210 DAFB) and SH cultivar ‘Xiacui’ (100 DAFB) at the earlier stage of ripeness were used for the transient transformation assay. *Agrobacterium* cultures carrying the *PpARF6* gene were injected into one side of the fruits, and the opposite side of the same fruits was infiltrated with empty vector. The infiltrated fruits were incubated in the growth chamber at 25°C with 60–70% relative humidity and a 12-h light/12-h dark photoperiod.

Three days after infiltration for ‘Dongxuemitao’ or six days after infiltration for ‘Xiacui’, flesh tissues surrounding the injection sites were collected, and each fruit was treated as an independent replicate. Five biological replicates were conducted for each treatment. All primers used for the vector construction in this study are available in Supplementary Data Table S2.

### Dual-luciferase reporter assay

Dual-luciferase reporter (LUC/REN) assay was performed based on a previous report [63]. The full-length CDSs of *PpARF6* and *PpEIL2/3* were individually ligated into the pRI-AN vector driven by the CaMV 35S promoter as effectors. The 2.0-kb promoter fragments of *PpACS1* and *PpACO1* containing ARF (TGTCNN) and EIL (ACATACAT) binding sites were cloned into the pGreenII0800-LUC vector as reporters. The transformed *Agrobacterium* cells were collected and suspended in infiltration buffer (10 mM MgCl_2_, 40 μM acetosyringone). *Agrobacterium* cultures containing the effector of *PpARF6* or *PpEIL2/3* and with the reporters and pSoup-p19 were mixed at a ratio of 9:1:1 (v/v/v). Mixed cultures were infiltrated into 4-week-old *Nicotiana benthamiana* leaves. *Agrobacterium* cultures carrying the pRI-AN empty vector were infiltrated as control. Three days after infiltration, firefly LUC and REN activities were measured using a dual-luciferase reporter assay system (Promega Biotech Co., Ltd, Beijing, China).

### Firefly luciferase complementation imaging assay

The firefly luciferase complementation (NC-LUC) assay was conducted as reported by Zhou *et al*. [44]. Briefly, the entire CDSs of *PpEIL1/2/3* were inserted into pCambia1300-cLUC vector [64], while whole CDSs of *PpARF6* and *PpEBF1/2* (without stop codon) were inserted into pCambia1300-nLUC [66]. *Agrobacterium* cultures harboring *PpEIL1/2/3* were mixed with those harboring either *PpARF6* or *PpEBF1/2* in a 1:1 (v/v) ratio and the mixture was infiltrated into 4-week-old *N. benthamiana* leaves. After 3 days of infiltration, the luminescence images of firefly luciferase activity were captured using an Image Quant LAS 4000 chemiluminescence imaging machine (GE Healthcare).

### Yeast one-hybrid, two-hybrid and three-hybrid assays

The Y1H, Y2H, and Y3H assays were carried out using Matchmaker® Gold Yeast Hybrid System as described in manufactures’ instructions. For Y1H, promoter fragments of *PpACS1* or *PpACO1* were ligated into the pAbAi plasmid to construct pAbAi-bait vectors. Whole coding sequences of *PpARF6* or *PpEIL2/3* were ligated to the pGADT7 plasmid to generate AD-prey vectors. The pAbAi-bait plasmid was digested with restriction enzyme BstBI and transformed into the Y1H Gold strain. The resulting transformants were screened on a synthetic defined (SD) medium lacking Ura (SD/−Ura) without or with the addition of AbA. Then, the AD-prey plasmids were individually introduced into the Y1H bait strains harboring pAbAi-bait vectors. Yeast cells carrying both AD-prey and pAbAi-bait vectors were dissolved in ddH_2_O and diluted to a series of concentrations, 0.1, 0.01, and 0.001. Interactions of PpARF/PpEIL with the promoters of *PpACS1/PpACO1* were estimated according to the growth rate of the co-transformants on SD−Ura/Leu medium containing AbA.

For Y2H, full CDSs of *PpEIL1/2/3* were ligated to the pGADT7 vector, while entire CDSs of *PpEBF1/2* were ligated to the pGBKT7 vector. Since the N-terminal of ARF6 is known to have self-transcriptional activation activity [65], its C-terminal containing conserved protein interaction domains, termed PpARF6^CTD^, was cloned into the pGBKT7 vector. The fusion vectors were co-introduced into the Y2H Gold strain and transformed cells were grown on SD/−Trp−Leu (SD/−2) medium at 30°C. Single colonies were suspended in ddH_2_O, and the suspended yeast cells were grown on plates of SD/−2, SD/−4, and SD/−4 containing 20 ng/ml X-α-gal and 200 ng/ml AbA at 30°C for 3–5 days. The interaction between EIL2/3-AD and ARF6/EBF1/EBF2-BD was assayed according to the color of yeast on SD/−4 containing X-α-gal.

For Y3H, the full CDSs of *PpEBF1/2* and *PpARF6* were inserted into the MCS I and MCS II sites of the pBridge plasmid, respectively, resulting in a pBridge-PpEBF1/2-PpARF6 construct. PpEBF1/2 was fused to the GAL4 binding domain, while PpARF6 was used as a ‘bridge’ protein and expressed only in the absence of methionine (Met). The pBridge-EBF1/2-ARF6 and PpEIL2/3-AD constructs were co-introduced into the Y2H strain, and co-transformed cells were grown on SD/−2 medium. The interaction between PpEBF1/2 and PpEIL2/3 was estimated by the survival of co-transformed cells lacking the *PpARF6* expression on SD/−4 medium, whereas the induction of *PpARF6* on SD/−5 was used to evaluate its impact on the interaction between PpEBF1/2 and PpEIL2/3. Photographs were taken 5 days after incubation at 30°C.

### Subcellular localization assay

*Agrobacterium* cells harboring either the *Super:PpARF6-GFP* or *Super:PpEIL-GFP* constructs mentioned above were suspended in infiltration buffer (20 g/l sucrose, 10 mM MgCl_2_, 50 mM MES, 20 μM acetosyringone, pH 5.8) with an OD_600_ of 1.0. The suspension was mixed with *Agrobacterium* cells carrying a tonoplast marker and the mixture was injected into leaves of 4-week-old transgenic *N. benthamiana* plantlets that carried a red fluorescent nuclear marker (Nucleus-RFP). Three days after infiltration, fluorescence signals were observed under a fluorescence microscope.

### Tomato stable transformation

Seeds of cultivated tomato (*Solanum lycopersicum*) ‘Alisa Craig’ (AC) were sterilized with 75% ethanol (2 min) and saturated sodium phosphate (20 min). The sterilized seeds were plated on half-strength Murashige and Skoog medium (½ MS) in a growth chamber maintained at 25°C under a 12-h light/12-h dark photoperiod until the cotyledons emerged. *Agrobacterium*-mediated cotyledon explant transformation was carried out as described by Li *et al*. [66]. After removing the distal and proximal parts of the cotyledon, the middle part was submerged in *Agrobacterium* cultures (OD_600_ = 1.8–2.0) harboring the above-mentioned *Super:PpARF6-GFP* construct for 0.5 h at room temperature. After removal of excess *Agrobacterium* suspension, the explants were placed on a Petri dish containing MS medium and incubated in the growth chamber at 25°C under darkness for 48 h. Then, the explants were transferred onto MS medium supplemented with 30 mg/l hygromycin and stored in the growth chamber at 25°C under a 16-h light/8-h dark photoperiod. When shoots were 2–4 cm in length, they were excised and placed on rooting medium containing 30 mg/l hygromycin. When roots were established, the transformants were planted in pots with soil. To validate the success of transformation, transcripts of *PpARF6* in hygromycin-resistant transgenic lines were determined by RT–qPCR. Finally, the positive seedlings were transferred to the greenhouse to let the plants grow.

### Measurement of total chlorophyll and carotenoid contents

The total chlorophyll and carotenoid content was measured based on a reported method [67] with minor modifications. Briefly, 0.2 g of tomato flesh powder was extracted in 5 ml 80% acetone in darkness for 24 h until the samples were completely decolorized. The supernatants were collected (4000 g, 10 min, 25°C) and the absorption spectra at 470, 646, and 663 nm were measured. The total chlorophyll and carotenoid content was calculated according to the equations reported in a previous study [68].

### Measurement of 1-aminocyclopropane-1-carboxylic acid

The extraction and measurement of ACC was conducted by MetWare (http://www.metware.cn/). Briefly, 0.05 g of tomato flesh powder was extracted in 0.5 ml of methanol/formic acid/water (15:4:1, v/v/v). The mixture was oscillated at 25°C for 3 min and then placed in an ice-water bath for ultrasonic crushing for 20 min. The supernatant was collected (12 000 g, 10 min, 4°C) and mixed with 20 μl of ACC internal standard intermediate solution (10 μg/ml). The mixture was evaporated to dryness using a nitrogen blower. Then, the sample was oximated in 100 μl methoxamine salt pyridine (0.015 g/ml) at 37°C for 2 h, and 100 μl of BSTFA–1% TMCS was added at 37°C for 0.5 h. The final solution was diluted 2 times with *n*-hexane and then subjected to measurement using the Agilent 7890B/G7000D GC–MS/MS system. The ACC content was expressed in nanomoles per gram.

### Statistical analysis

All experiments were performed with at least three biological replicates. Statistical analysis was conducted using SigmaPlot 12.5 and statistical significance was estimated based on Student’s *t*-test.

## Supplementary Material

Web_Material_uhad158

## Data Availability

All the data supporting the findings of this study are available in the paper and supplementary data.
