# Supplementary material for: PpARF6 acts as an integrator of auxin and ethylene signaling to promote fruit ripening in peach
Source: Hortic Res. 2023 Jul 31;10(9):uhad158. doi: 10.1093/hr/uhad158 (PMC10500152; doi:10.1093/hr/uhad158)
Supplement: Web_Material_uhad158 [file web_material_uhad158.zip › Submit to HR SupplementaryA Data.docx]

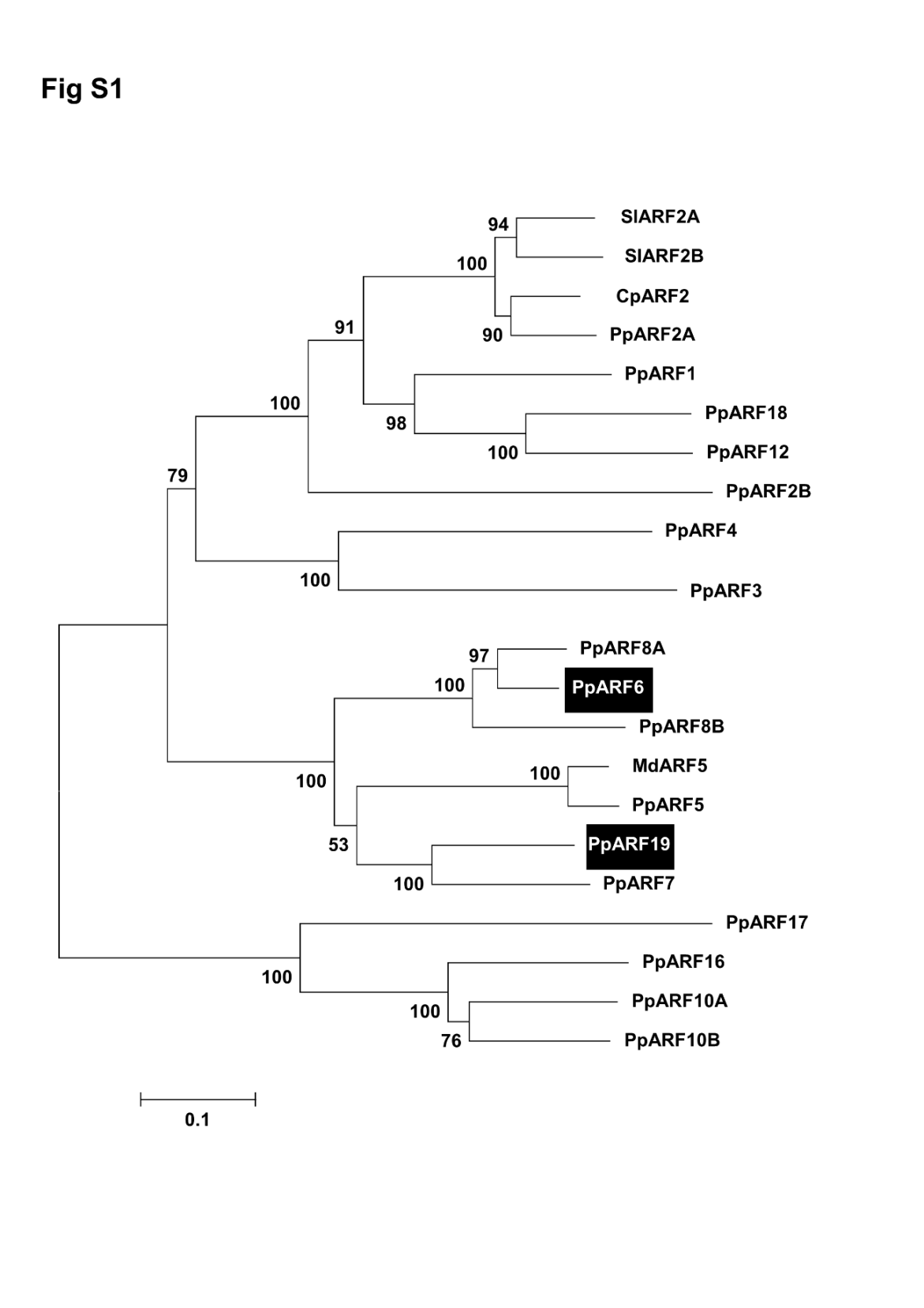


**Figure S1.** **Phylogenetic tree derived from amino acid sequences of ARF transcription factors in peach and ripening-related** **ARFs previously reported in other plant species**. PpARF6 and PpARF19 that are highly expressed in peach fruits during ripening stages are highlighted in black background.


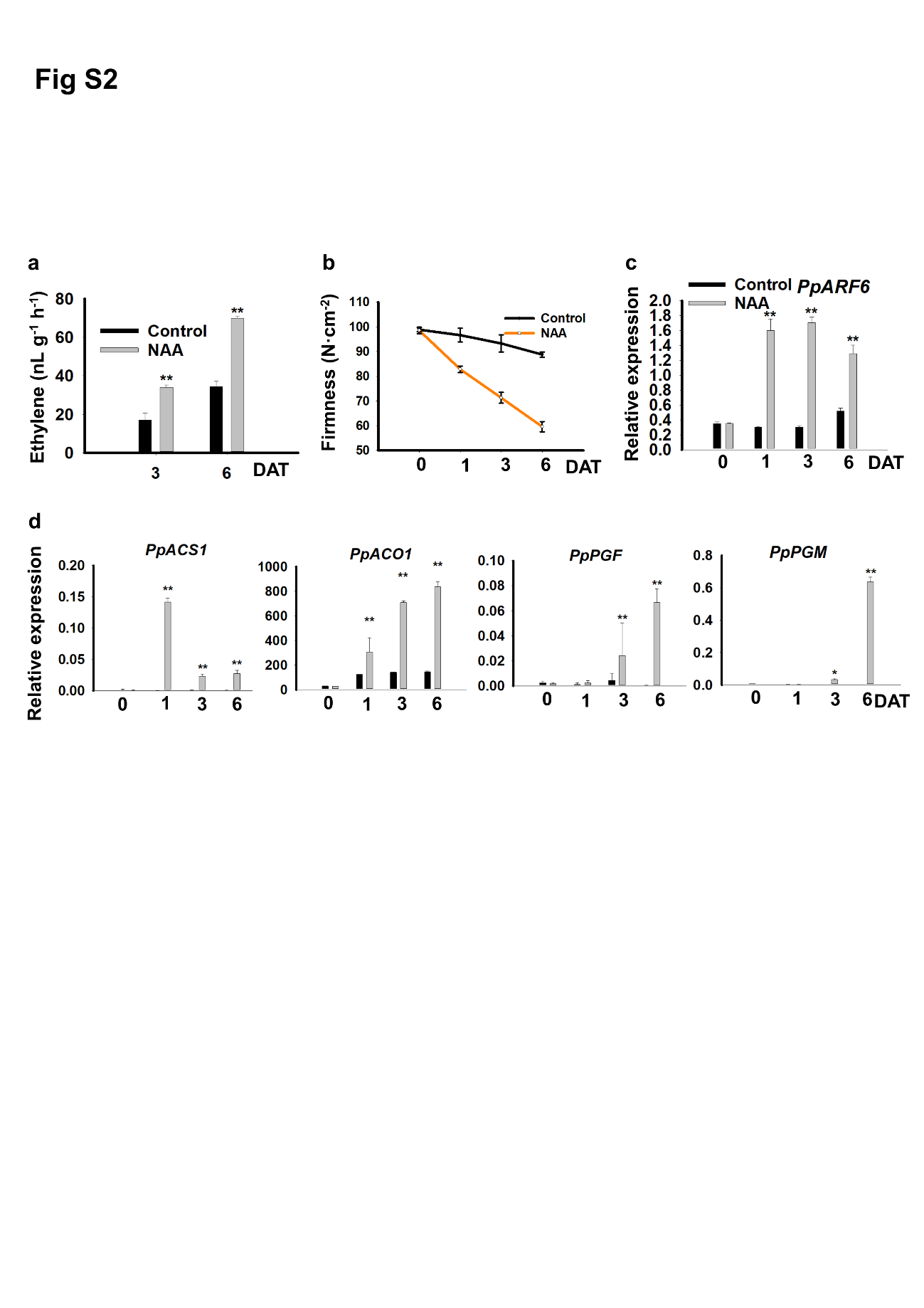


**Figure S2.** **Influence of NAA treatment on fruit ripening of SH peach cultivar ‘Xiacui’. (**a) Ethylene content in peach fruits after treated with NAA or ddH_2_O. (b) Changes in fruit firmness after NAA treatment. (c and d) Expression profiles of *PpARF6*, ethylene biosynthetic genes and softening-related genes in peach fruits after treated with NAA or ddH_2_O.


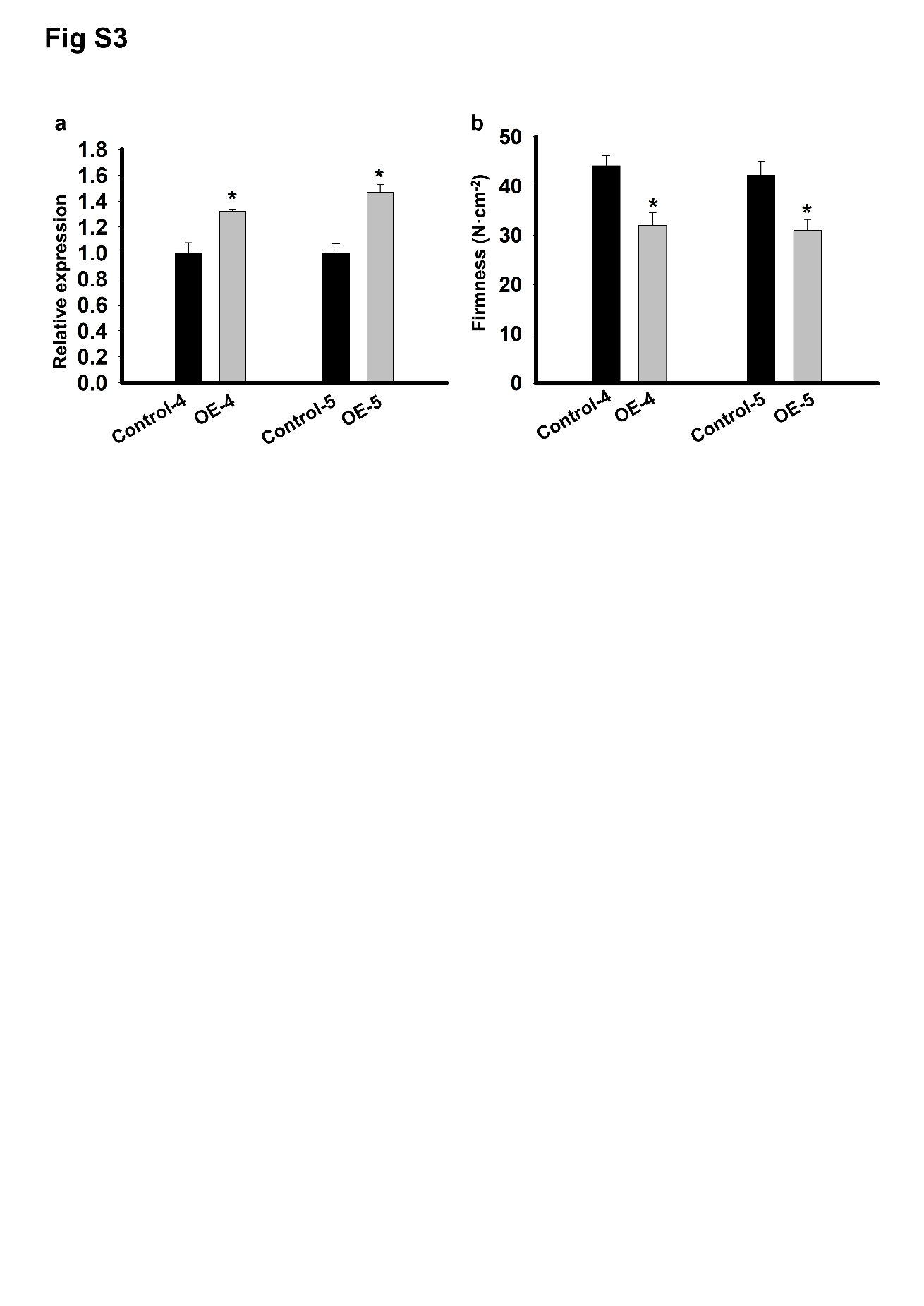


**Figure S3. Transient overexpression of *PpARF6* in peach** **fruits of melting flesh cultivar ‘Dongxuemitao’.** (a) Relative expression of *PpARF6* in flesh tissues around the sites infiltrated with *PpARF6* or empty vector (control). (b) Firmness in flesh tissues around the sites infiltrated with *PpARF6* or empty vector (control).


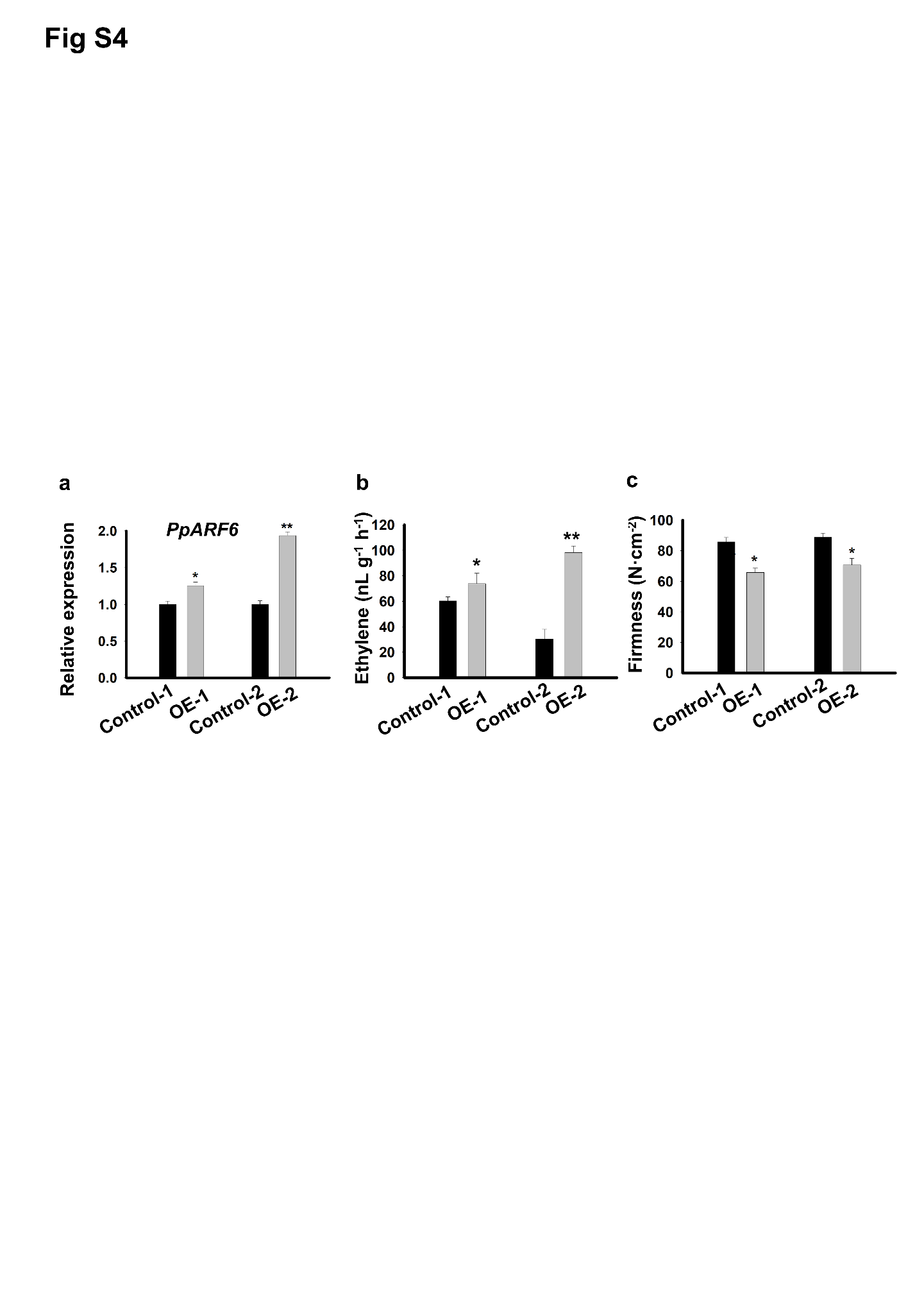


**Figure S4. Transient overexpression of *PpARF6* in peach fruits of SH variety ‘Xiacui’.** (a) Relative expression of *PpARF6* in flesh tissues around the sites infiltrated with *PpARF6* or empty vector (control). (b) Ethylene content in flesh tissues around the sites infiltrated with *PpARF6* or empty vector (control). (c) Firmness in flesh tissues around the sites infiltrated with *PpARF6* or empty vector (control).


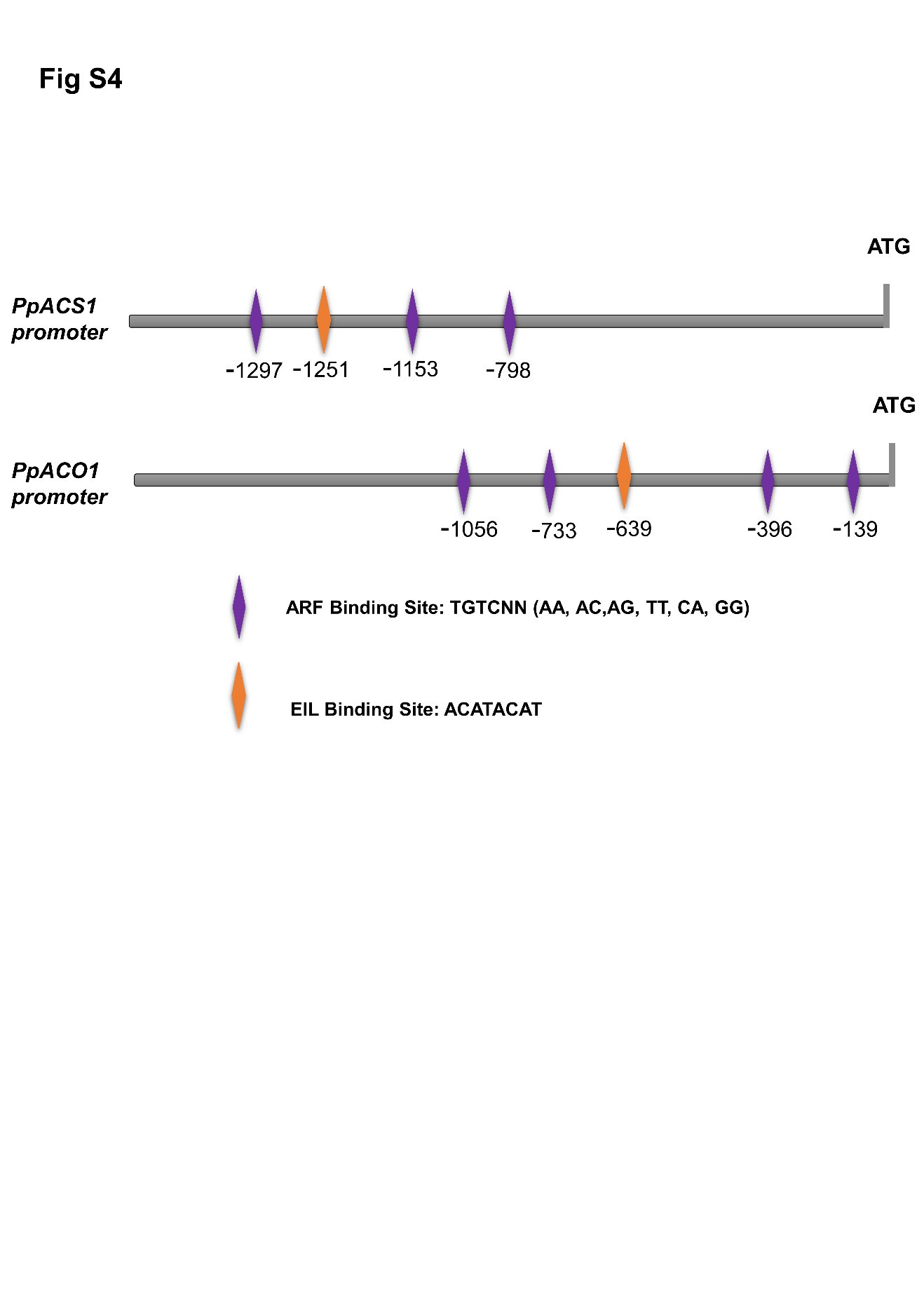


**Figure S5. Schematic diagram of ARF and EIL binging sites in the promoters of *PpACS1* and *PpACO1*.**


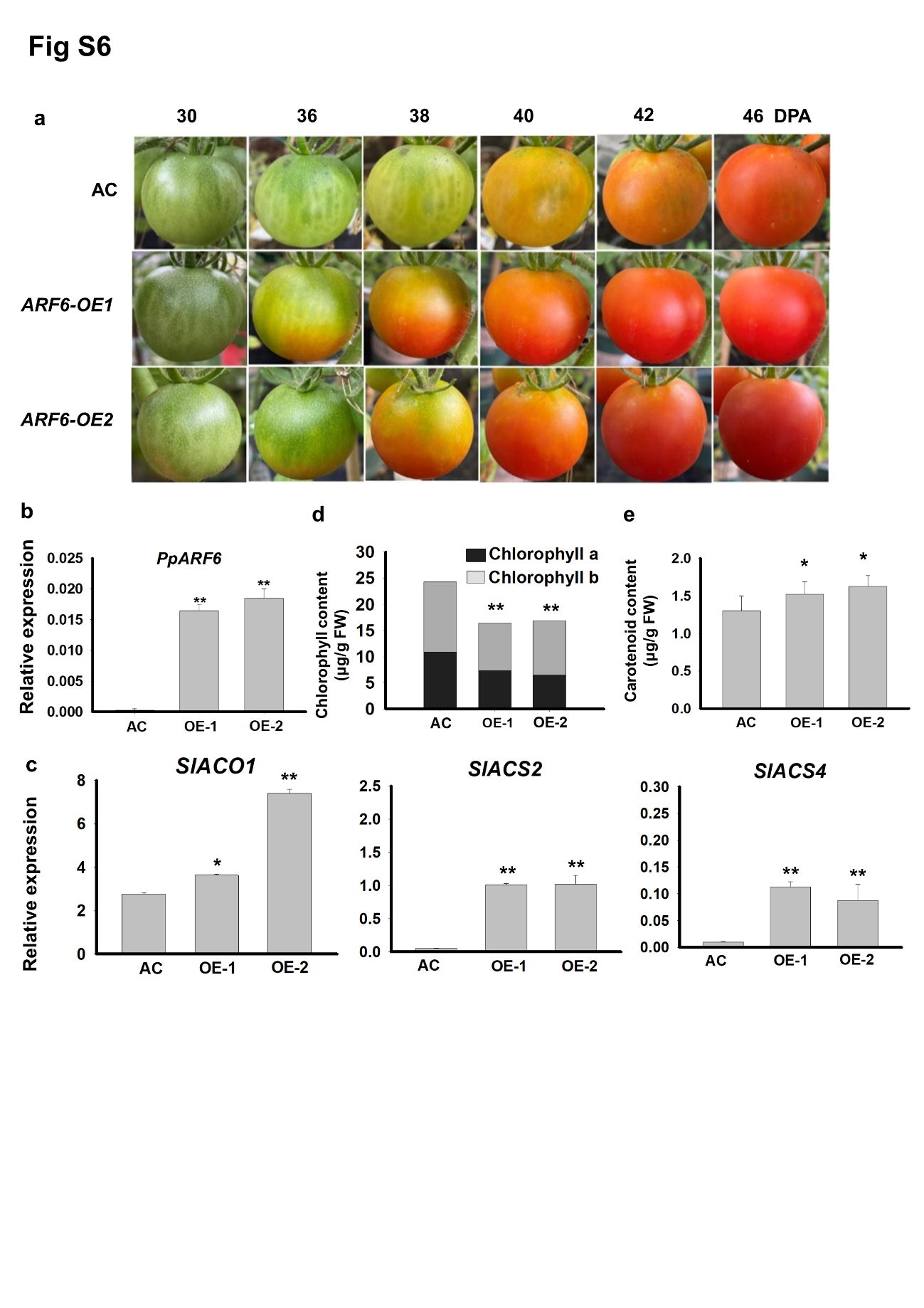


**Figure S6. Functional analysis of the regulatory role of *PpARF6* in fruit ripening behavior via its ectopic overexpression in tomato.** (a) The ripening behavior of transgenic tomato fruits overexpressing *PpARF6* and the non-transgenic tomato fruits of ‘AC’ at different stages of ripening. Fruit ripening behavior was monitored at 30, 36, 38, 40, 42, and 46 days post-anthesis (DPA). (b) Expression of *PpARF6* in transgenic and ‘AC’ tomato fruits at 40 DPA. (c) Expression profiles of *SlACO1*, *SlACS2* and *SlACS4* in tomato fruits at 40 DPA. (d and e) Total contents of chlorophylls and carotenoids in tomato fruits at 40 DPA.


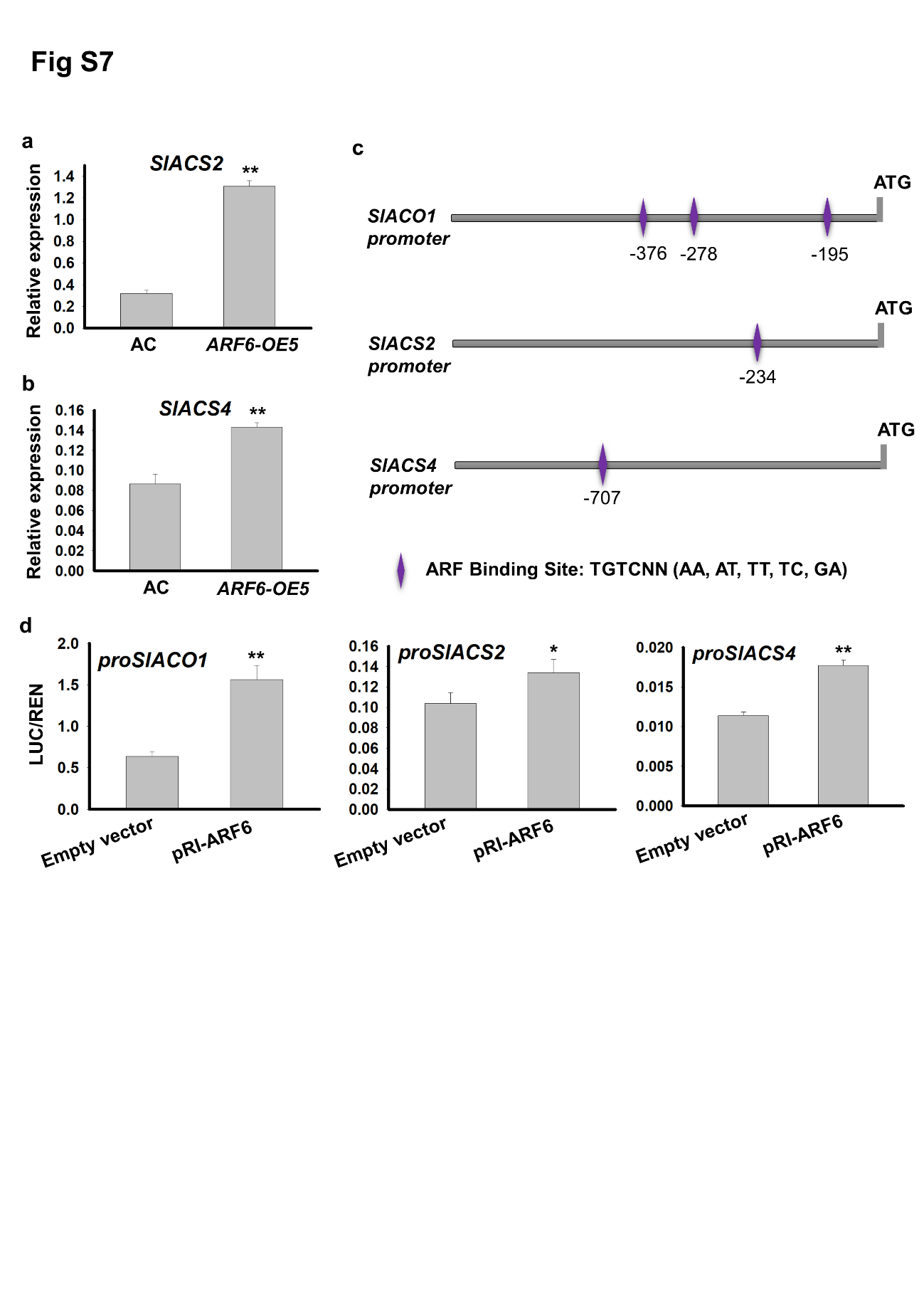


**Figure S7. Assay of activation of PpARF6 on the promoters of *SlACO1*, *SlACS2* and *SlACS4*. (**a and b) Expression of *SlACS2* and *SlACS4* in transgenic and non-transgenic tomato fruits at 10 days after breaker (DAB). (c) Schematic diagram of ARF binging sites in the promoters of *SlACO1*, *SlACS2* and *SlACS4*. **(**d) Assessment of activation of PpARF6 on the promoters of *SlACO1*, *SlACS2* and *SlACS4* using transient dual-luciferase reporter assay. The transcriptional activation activity was measured 3 days after infiltration, and each treatment contains at least three biological replicates.


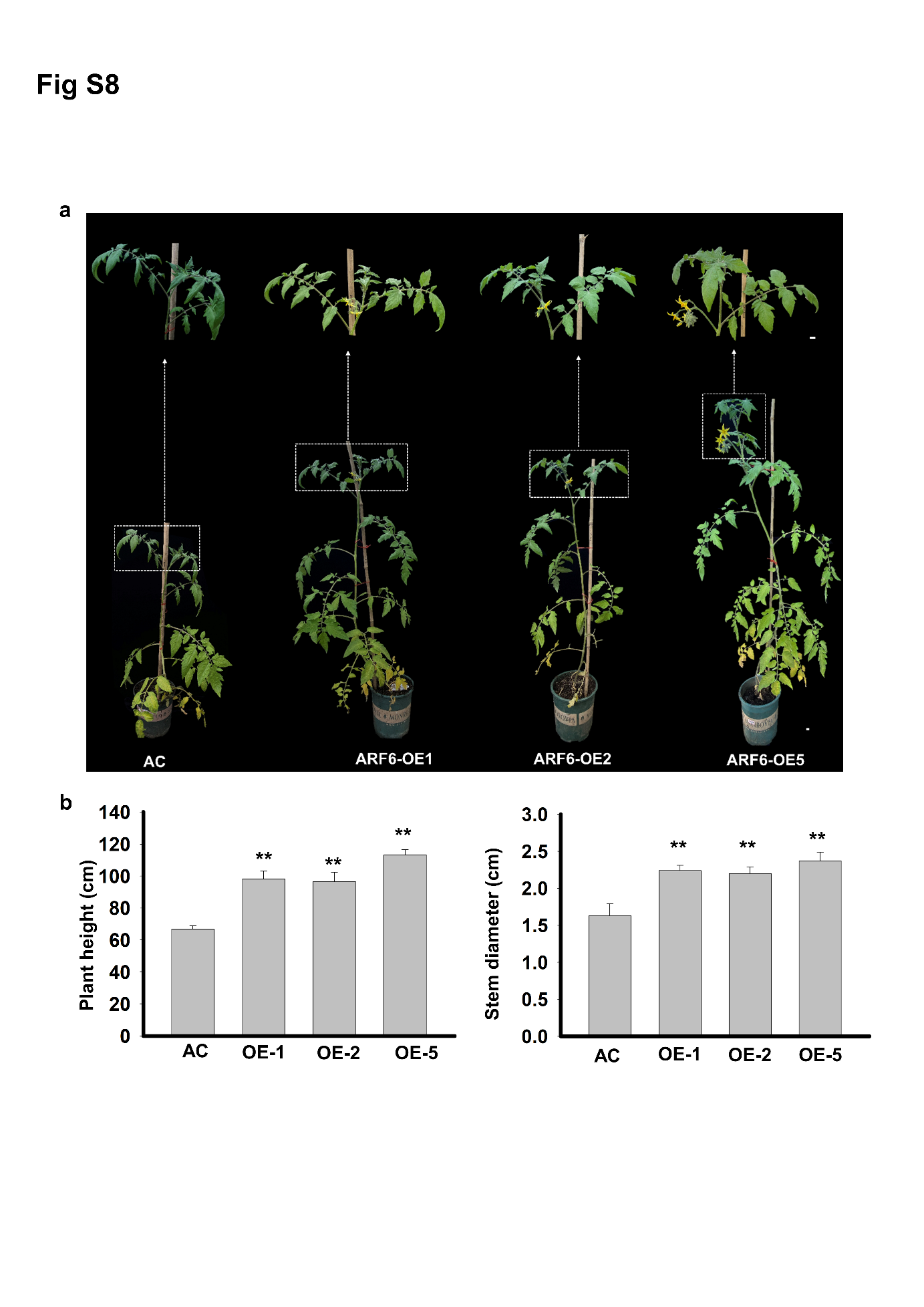


**Figure S8. Morphology (a) and growth parameters (b) of wild-type and** ***PpARF6* overexpressing transgenic plants.** The white bar indicates 1 cm. ** and * indicate statistical significance at *P* < 0.01 and *P* < 0.05, respectively, based on Student’s *t*-test.


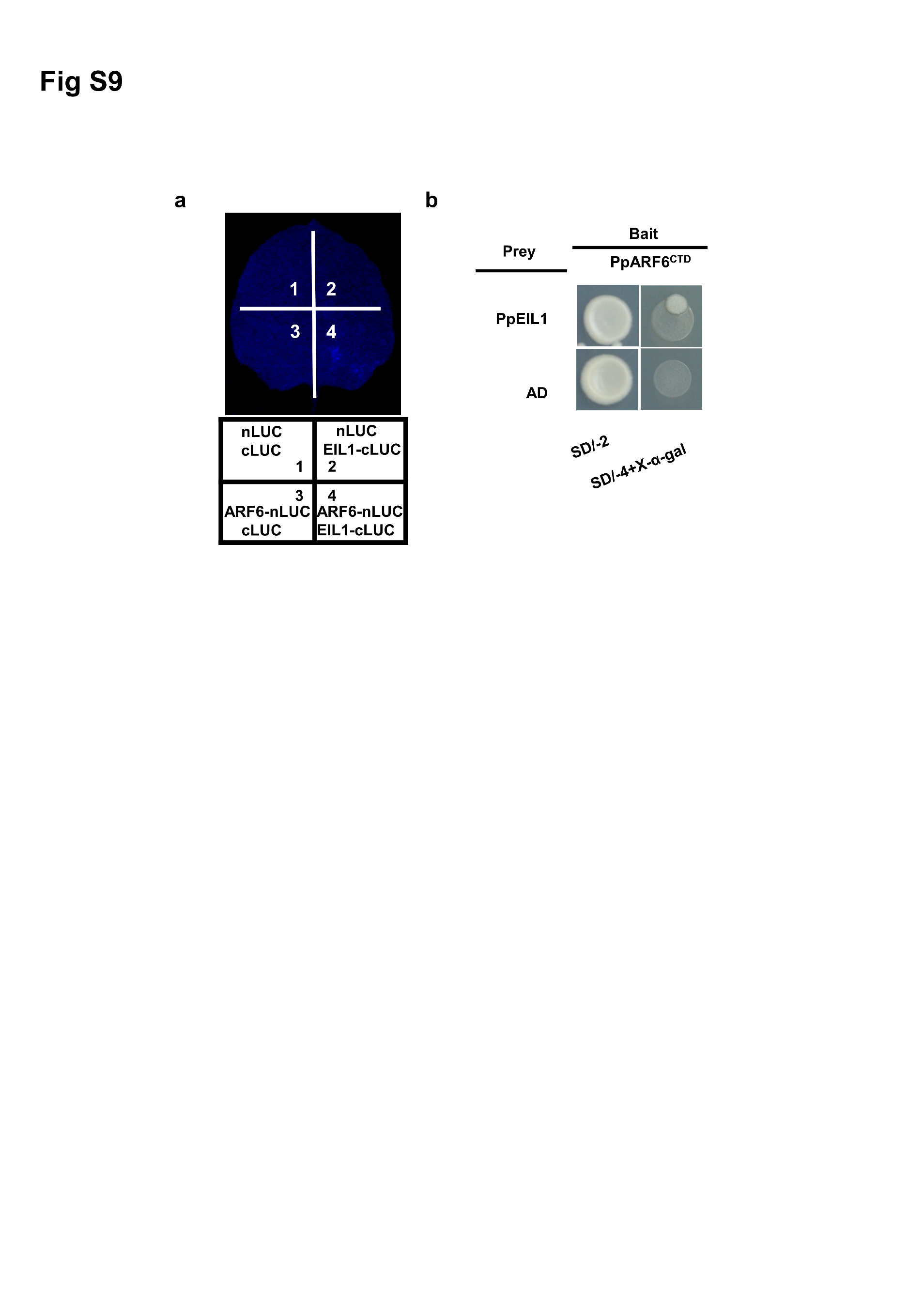


**Figure S9. Investigation of physical interaction between PpARF6 and PpEIL1 (*Prupe.6G018200*) using firefly NC-LUC assay (a) and Y2H assay (b).**


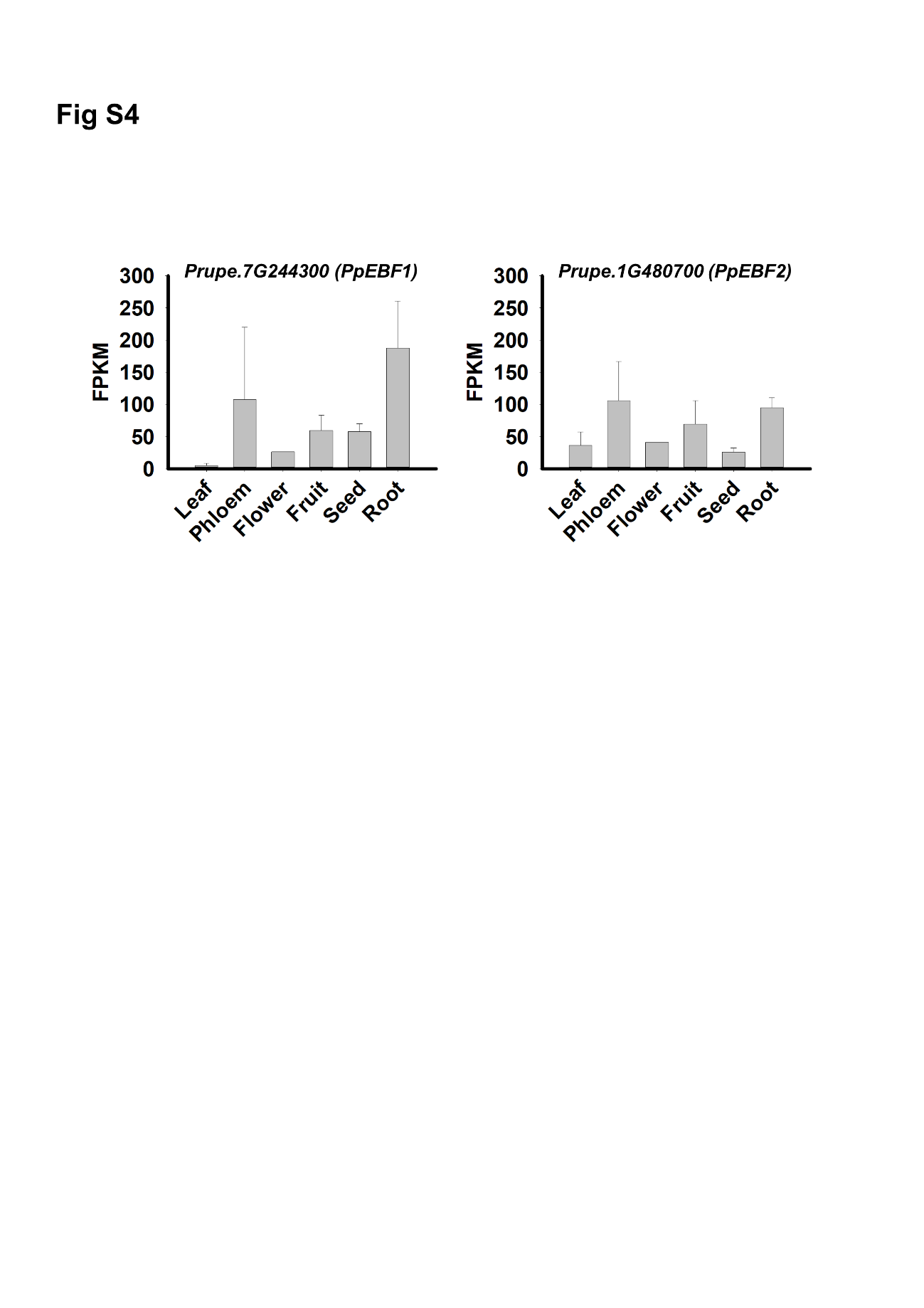


**Figure S10. Gene expression profile of** ***PpEBF1* (*Prupe.7G244300*) and** ***PpEBF2* (*Prupe.1G480700*) in the phloem tissue of peach and other organs, including flower, fruit, root and seed.** *PpEBF1* and *PpEBF2* are highly expressed in ripe fruit.


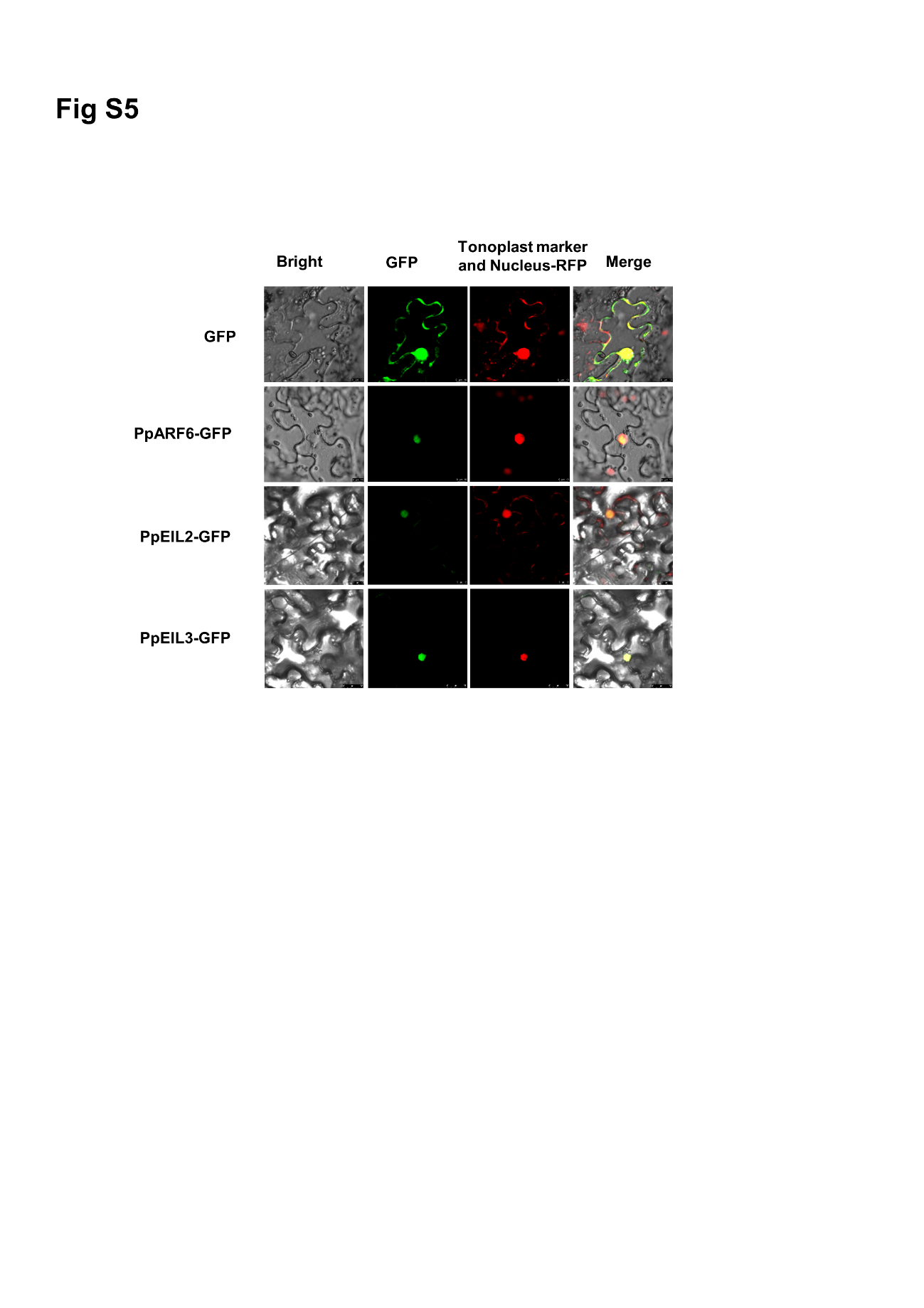


**Figure S11.** **Analysis of subcellular localization of PpARF6 and PpEIL2/3 in tobacco leaves.** The PpARF6-, PpEIL2/3-pSuper1300GFP fusion protein, and tonoplast marker were mixed and transiently expressed in *N*. *benthamiana* leaves. Scale bars, 10 μm. PpEIL2 (*Prupe.2G058400*), PpEIL3 (*Prupe.2G058500*).


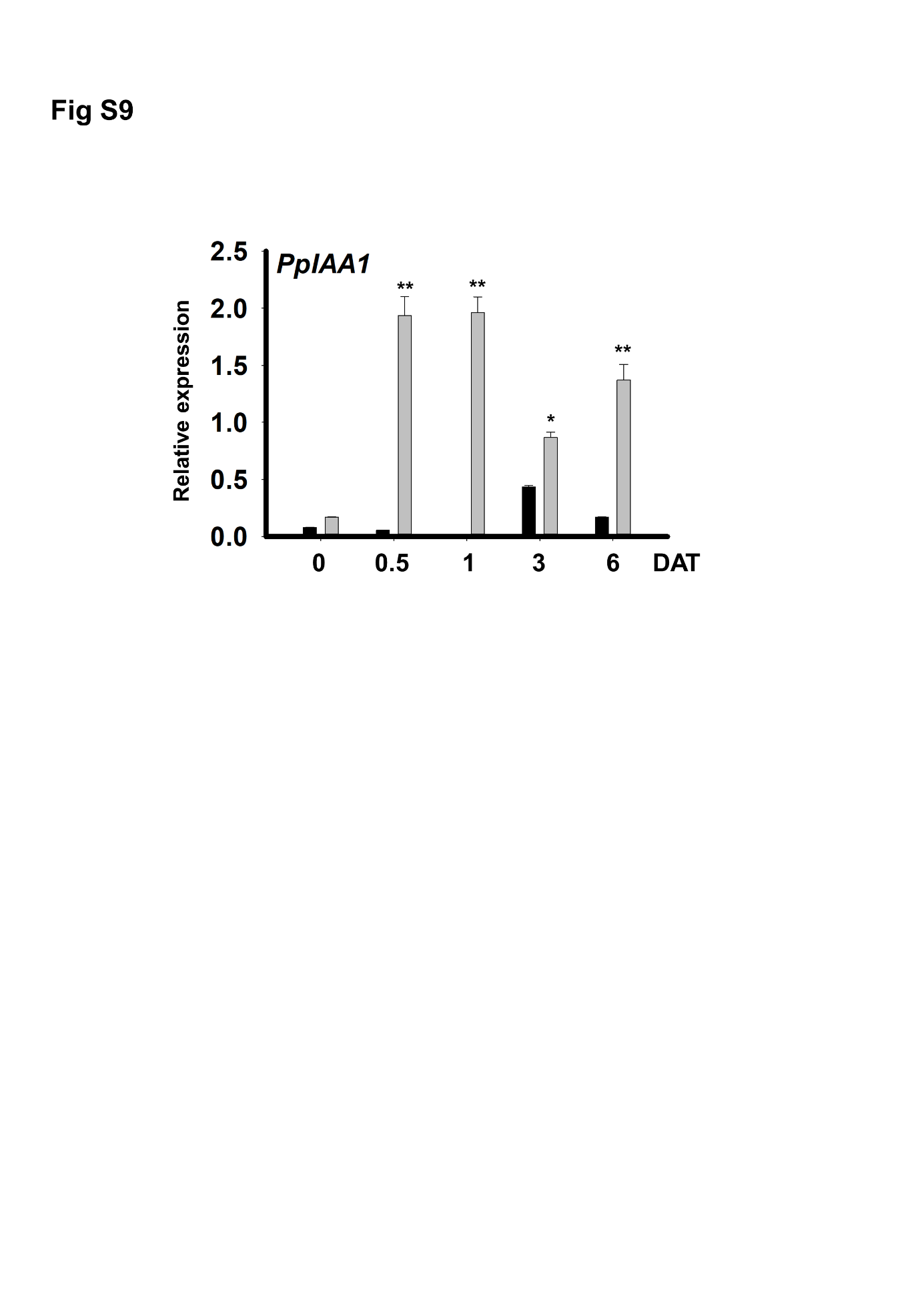


**Figure S12.** **Expression of *PpIAA1* in fruits treated with NAA or ddH_2_O that are highlighted in gray and back colors, respectively**. Data represent average values of three biological replicates. Statistical significance was estimated based on student’s *t*-test. **, *P* < 0.01, * *P* < 0.05.


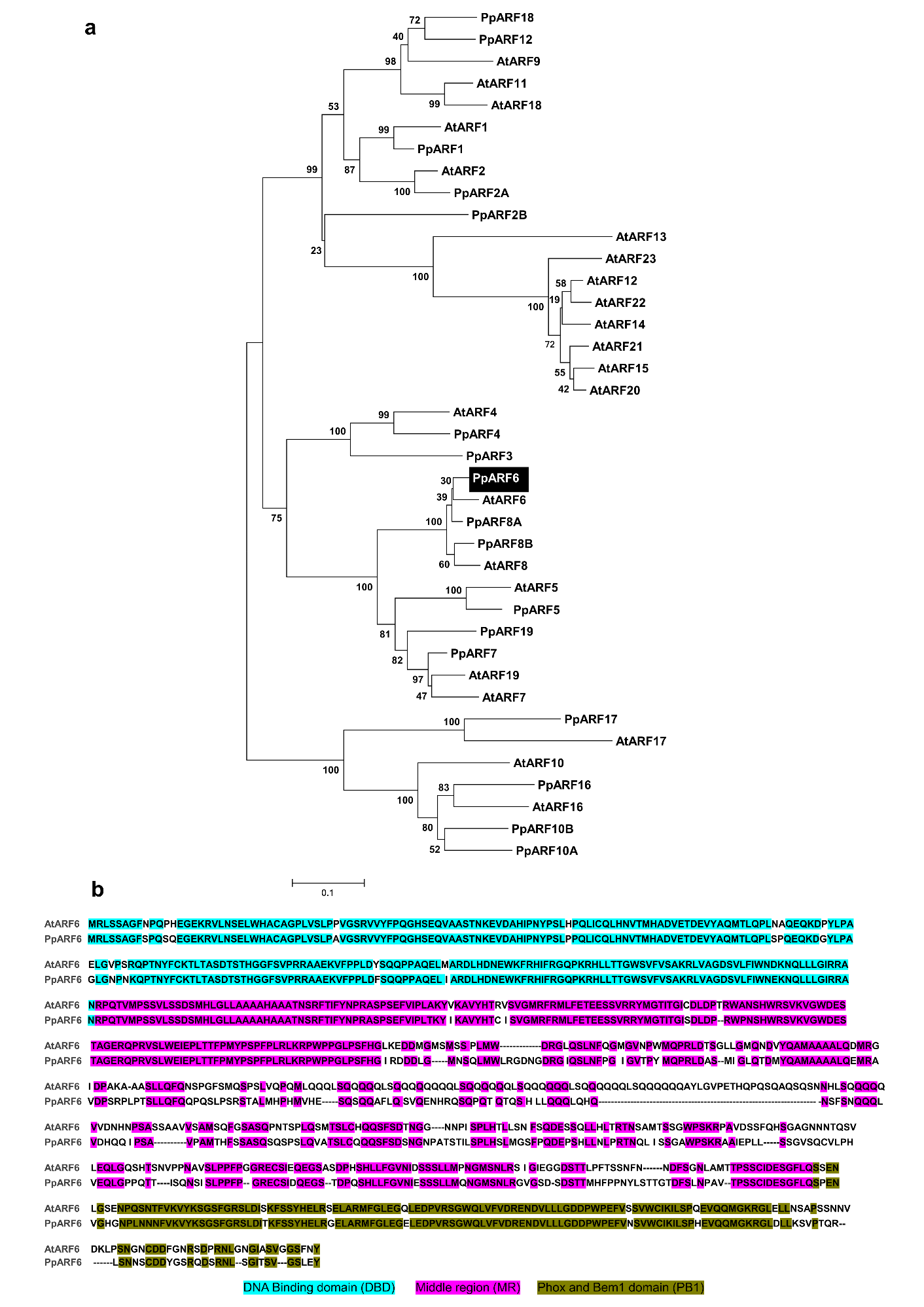


**Figure S13.** **Comparison of ARF transcription factors (TFs) between peach and *Arabidopsis thaliana***. (a) Phylogenetic tree derived from amino acid sequences of ARF TFs in peach and *Arabidopsis***.** PpARF6 identified in this study are highlighted in black background. (b) Alignment of amino acid sequences of PpARF6 and AtARF6.

**Table S1** **Primers used for RT-qPCR analysis**

| Gene | Forward primer (5′ to 3′) | Reverse primer (5′ to 3′) | Phytozome/GenBank No. |
| --- | --- | --- | --- |
| *PpARF6* | GTCGGGCAGCTGAAAAAGTG | GCTGGCCACGAAAAATGTGT | *Prupe.4G085900* |
| *PpARF19* | TGCAACCACCTGCTCAAGAA | AGACAGAATCGCCAGCGAAA | *Prupe.1G065300* |
| *PpACS1* | AACCAAGGAAGCACTTGCAG | CACAAGGCTTTCGAGTGTGT | *Prupe.2G176900* |
| *PpACO1* | TACCAGGATCTGGTGGCAAG | GCCAGGGAAGTTCCTGTTTG | *Prupe.3G209900* |
| *PpPGF* | ATCCCTAAACAGCCAAATCTTCCAT | TGCCATCGGTGTTAGGGCTATTAC | *Prupe.4G262200* |
| *PpPGM* | TCCCTAATCAGCCAAATGTTCCAC | GCCATCGGTGTTAGGGCTGTTC | *Prupe.4G261900* |
| *PpIAA1* | GGCTGTTGGGATAGCTCCAA | GCTTGATCAGTACCATTCATTTCAT | *Prupe.7G234800* |
| *SlACO1* | GCCAAAGAGCCAAGATTTGA | TTTTTAATTGAATTGGGATCTAAGC | *Solyc07g049530* |
| *SlCNR* | GCCAAATCAAGCAATGATGA | TCGCAACCATACAGACCATT | *Solyc02g077920* |
| *SlPL* | GCGATCAGGAGTTAGAACTGG | AATCCCCTTTTGCTTTGGTT | *Solyc03g111690* |
| *SlPSY1* | CCCTGTATGGGCATCTTTGG | TGATTTGCTCACATATGCTCTCTTT | *Solyc03g031860* |
| *SlPG2* | AGAATTTGCTCATGATTTTCAAGC | TGTTTTTCCATCACCCTTAGCTC | *Solyc10g080210* |
| *SlACTIN2* | TTGCTGACCGTATGAGCAAG | GGACAATGGATGGACCAGAC | *Solyc11g005330* |
| *PpTEF2* | GGTGTGACGATGAAGAGTGATG | TGAAGGAGAGGGAAGGTGAAAG | *JQ732180* |

**Table S2 Primers used for vector construction**

| Name | Forward primer Sequence (5′ to 3′) | Reverse primer Sequence (5′ to 3′) |
| --- | --- | --- |
| pSuper1300-GFP-ARF6 | ATACTAGTGGATCCGGTACCATGAGGCTCTCATCTGCTGG | CCCTTGCTCACCATGGTACCATACTCGAGTGACCCCACG |
| pSuper1300-GFP-EIL2 | ATACTAGTGGATCCGGTACCATGGGGATCTTTGAAGAAATGGG | CCCTTGCTCACCATGGTACCGATCCAAAATGCATCTTGCTTC |
| pSuper1300-GFP-EIL3 | ATACTAGTGGATCCGGTACCATGGGGATCTTTGAGGAAATGG | CCCTTGCTCACCATGGTACCTGGGAACCAAAAGGATGCATC |
| pRI-AN-ARF6 | ACCCCGGGGGTACCGGATCCATGAGGCTCTCATCTGCTGG | TTGATTCAGAATTCGGATCCTCAATACTCGAGTGACCCCACG |
| pRI-AN-EIL2 | ACCCCGGGGGTACCGGATCCATGGGGATCTTTGAAGAAATGGG | TTGATTCAGAATTCGGATCCTCAGATCCAAAATGCATCTTGCTTC |
| pRI-AN-EIL3 | ACCCCGGGGGTACCGGATCCATGGGGATCTTTGAGGAAATGG | TTGATTCAGAATTCGGATCCTTATGGGAACCAAAAGGATGCATC |
| PGreen-LUC-PpACO1 | gggcgaattgggtaccTTGGCTAAGTCACATCAAGGCAT | gctctagaactagtggatccCTCTCTCTCTCTCTTTGTGTGT |
| PGreen-LUC-PpACS1 | gggcgaattgggtaccTTATGTGCCATGATGTTCCACAAAGTC | gctctagaactagtggatccTTTCTTGGTTCCAAAGAATACTCACACAC |
| AbAi- PpACO1 | tgaattcgagctcTTGGCTAAGTCACATCAAGGCATGC | agcacatgcctcgagGCCAATCTAATATCCTGGATTAAC |
| AbAi- PpACS1 | tgaattcgagctcTTATGTGCCATGATGTTCCACAAAGTC | agcacatgcctcgagTTTCTTGGTTCCAAAGAATACTCACACAC |
| AD- PpARF6 | ccatggaggccagtgaattcATGAGGCTCTCATCTGCTGG | agctcgagctcgatggatcc TCAATACTCGAGTGACCCCAC |
| AD-EIL1 | ccatggaggccagtgaattcATGGGTGACGTTGAAGAGG | agctcgagctcgatggatccTTATGCCGCAAAGTATTCCATC |
| AD-PpEIL2 | ccatggaggccagtgaattcATGGGGATCTTTGAAGAAATGGG | agctcgagctcgatggatccTCAGATCCAAAATGCATCTTGCTTC |
| AD-PpEIL3 | ccatggaggccagtgaattcATGGGGATCTTTGAGGAAATGG | agctcgagctcgatggatccTTATGGGAACCAAAAGGATGCATC |
| BD-PpARF6^CTD^ | TGGCCATGGAGGCCGAATTCCAAACTTCTCAGCAACATGTCAGAGATGG | CGCTGCAGGTCGACGGATCCTTAAGGAAGTAGCTGAGACTTCCCCTCTC |
| BD-EBF1 | TGGCCATGGAGGCCGAATTCATGCCTGCCCTCGTTAATTACAG | CGCTGCAGGTCGACGGATCCTTAAGCCAGGATATCACATCTCC |
| BD-EBF2 | TGGCCATGGAGGCCGAATTCATGTCAAAGCTCCTTGGTTTCG | CGCTGCAGGTCGACGGATCCCTAGGAAAGGATATCACAACGCC |
| nLUC-ARF6 | cgggggacgagctcggtaccATGAGGCTCTCATCTGCTGG | gcgtacgagatctggtcgacATACTCGAGTGACCCCACG |
| nLUC-EBF1 | cgggggacgagctcggtaccATGCCTGCCCTCGTTAATTACAG | gcgtacgagatctggtcgacAGCCAGGATATCACATCTC |
| nLUC-EBF2 | cgggggacgagctcggtaccATGTCAAAGCTCCTTGG | gcgtacgagatctggtcgacGGAAAGGATATCACAACGC |
| cLUC- PpEIL1 | acgcgtcccggggcggtaccATGGGTGACGTTGAAGAGG | cgaaagctctgcaggtcgacTTATGCCGCAAAGTATTCCATC |
| cLUC- PpEIL2 | acgcgtcccggggcggtaccATGGGGATCTTTGAAGAAATGGG | cgaaagctctgcaggtcgacTCAGATCCAAAATGCATCTTGCTTC |
| cLUC- PpEIL3 | acgcgtcccggggcggtaccATGGGGATCTTTGAGGAAATGG | cgaaagctctgcaggtcgacTTATGGGAACCAAAAGGATGCATC |
| pBridge-EBF1 | TGACTGTATCGCCGGAATTCATGCCTGCCCTCGTTAATTACAGTGG | GGCTGCAGGTCGACGGATCCTTAAGCCAGGATATCACATCTCCACAAGCTC |
| pBridge-EBF2 | TGACTGTATCGCCGGAATTCATGTCAAAGCTCCTTGGTTTCGCTG | GGCTGCAGGTCGACGGATCCCTAGGAAAGGATATCACAACGCCATAGCTG |
| pBridge-EBF-ARF6 | AGAGAAAGGTGGCGGCCGCAATGAGGCTCTCATCTGCTGGTTTTAGTC | GGAGATCAGCCCGAAGATCTTCAATACTCGAGTGACCCCACGG |

**Table S3** **FPKM values used for the analysis of heat map shown in Fig. 1a.** S1 to S4 represent four phases of fruit development. Each stage contains three biological replicates.

| Gene ID | S1 | | | S2 | | | S3 | | | S4 | | |
| --- | --- | --- | --- | --- | --- | --- | --- | --- | --- | --- | --- | --- |
| *Prupe.1G065300* | 17.32 | 9.94 | 30.95 | 10.50 | 10.79 | 10.31 | 17.30 | 40.98 | 17.45 | 90.22 | 109.42 | 111.33 |
| *Prupe.7G194200* | 4.04 | 2.84 | 7.07 | 2.66 | 3.51 | 3.62 | 14.69 | 7.57 | 9.95 | 9.02 | 11.93 | 7.60 |
| *Prupe.1G368300* | 3.34 | 3.67 | 3.17 | 3.42 | 3.99 | 4.47 | 12.97 | 13.22 | 12.42 | 27.05 | 30.28 | 32.54 |
| *Prupe.3G182900* | 34.35 | 26.16 | 41.43 | 35.90 | 27.25 | 27.20 | 49.53 | 37.16 | 64.85 | 28.21 | 31.42 | 30.53 |
| *Prupe.4G085900* | 32.75 | 39.01 | 28.11 | 23.33 | 23.14 | 32.57 | 158.36 | 92.87 | 188.45 | 138.60 | 132.19 | 110.78 |
| *Prupe.5G143100* | 107.73 | 101.11 | 71.08 | 47.63 | 47.07 | 44.31 | 32.56 | 33.02 | 42.07 | 21.68 | 21.85 | 18.94 |
| *Prupe.6G097700* | 5.20 | 3.67 | 6.43 | 2.34 | 2.69 | 5.01 | 15.49 | 17.32 | 16.31 | 19.29 | 27.77 | 12.21 |
| *Prupe.4G053800* | 4.64 | 4.04 | 3.54 | 4.72 | 3.24 | 3.81 | 6.45 | 8.15 | 11.07 | 3.20 | 2.04 | 1.16 |
| *Prupe.6G102800* | 2.36 | 6.09 | 1.86 | 2.48 | 2.11 | 1.04 | 10.45 | 8.45 | 13.37 | 4.40 | 1.97 | 2.94 |
| *Prupe.6G236200* | 5.64 | 5.21 | 3.76 | 4.90 | 7.37 | 3.93 | 10.21 | 8.81 | 10.26 | 8.66 | 4.69 | 5.64 |
| *Prupe.2G190400* | 9.30 | 7.66 | 4.21 | 2.56 | 2.10 | 1.89 | 0.98 | 1.32 | 1.50 | 0.52 | 0.35 | 0.44 |
| *Prupe.1G585200* | 13.63 | 16.29 | 8.51 | 13.21 | 15.22 | 14.42 | 16.97 | 16.99 | 24.07 | 16.68 | 13.98 | 11.06 |
| *Prupe.5G123400* | 1.11 | 1.27 | 0.92 | 0.31 | 0.15 | 0.28 | 0.32 | 0.76 | 0.48 | 0.68 | 0.42 | 0.21 |
| *Prupe.2G213000* | 3.82 | 1.21 | 2.36 | 0.63 | 0.57 | 0.49 | 4.14 | 1.64 | 2.74 | 3.09 | 1.71 | 1.68 |
| *Prupe.1G507000* | 0.70 | 0.74 | 0.78 | 0.82 | 0.71 | 0.81 | 2.56 | 2.30 | 3.66 | 4.17 | 6.16 | 5.01 |
| *Prupe.8G252300* | 38.84 | 4.23 | 72.08 | 8.10 | 8.71 | 22.81 | 1.31 | 2.01 | 0.44 | 1.53 | 1.21 | 1.71 |
| *Prupe.3G011800* | 53.32 | 30.43 | 39.78 | 33.81 | 26.02 | 31.16 | 39.56 | 40.05 | 29.05 | 20.55 | 28.83 | 24.78 |

**Table S4** **FPKM values used for profiling gene expression in different tissues of peach shown in Fig. 1b and Fig. 1c.** Each tissue consists of two biological replicates.

| Gene ID | Leaf | | Phloem | | | Flower | | | Fruit | | | Seed | | | Root | | |  |
| --- | --- | --- | --- | --- | --- | --- | --- | --- | --- | --- | --- | --- | --- | --- | --- | --- | --- | --- |
| *Prupe.4G085900 (PpARF6)* | 4.12 | 19.54 | | 11.32 | 18.95 | | 57.42 | 57.49 | | 25.17 | 33.29 | | 6.62 | 6.83 | | 33.05 | 13.18 | |
| *Prupe.1G065300 (PpARF19)* | 6.73 | 11.24 | | 17.59 | 25.31 | | 5.52 | 5.53 | | 14.75 | 19.4 | | 6.95 | 2.95 | | 8.01 | 13.62 | |
| *Prupe.7G244300 (PpEBF1)* | 7.25 | 2.87 | | 187.28 | 28.14 | | 26.06 | 26.1 | | 76.06 | 42.76 | | 66.43 | 49.25 | | 239.07 | 136.03 | |
| *Prupe.1G480700 (PpEBF2)* | 51.07 | 21.83 | | 62.65 | 149.04 | | 40.95 | 40.86 | | 94.98 | 43.62 | | 30.43 | 20.9 | | 83.9 | 106.04 | |
